# Genetic evidence substantiates transmission of *Trichinella spiralis* from one swine farm to another

**DOI:** 10.1186/s13071-021-04861-9

**Published:** 2021-07-09

**Authors:** Ewa Bilska-Zajac, Daniele Tonanzi, Edoardo Pozio, Miroslaw Rozycki, Tomasz Cencek, Peter C. Thompson, Benjamin M. Rosenthal, Giuseppe La Rosa

**Affiliations:** 1 National Veterinary Research Institute in Puławy, Al. Partyzantów 57, Puławy, Poland; 2 European Union Reference Laboratory for Parasites, Istituto Superiore Di Sanità, Rome, Italy; 3 United States Department of Agriculture, Agricultural Research Service, Animal Parasitic Diseases Laboratory, Beltsville, MD 20705 USA

**Keywords:** *Trichinella spiralis*, Microsatellite markers, Genetic structure, Outbreak, Pig, Rat, Wild boar, Molecular epidemiology

## Abstract

**Background:**

*Trichinella spiralis* ranks seventh in the risk posed by foodborne parasites. It causes most human cases of trichinellosis and is the most frequent cause of *Trichinella* outbreaks on pig farms and in wild boar, worldwide. Veterinary inspectors seek the source of outbreaks in hopes of limiting the spread. Established molecular tools are inadequate for distinguishing among potential *T. spiralis* infection sources because genetic variability in these zoonotic pathogens is limited in Europe. Microsatellite markers proved successful in tracing an outbreak of *T. britovi*, a related parasite harboring much more genetic variation. Here, we successfully employed microsatellite markers to determine the genetic structure of *T. spiralis* isolates from two pig outbreaks, discovering notable uniformity among parasites within each farm and discovering an epidemiological link between these two outbreaks.

**Methods:**

The individual larvae from five isolates of *T. spiralis* from two pig farms and from ten wild boars were genotyped using nine microsatellite markers to examine their genetic structure.

**Results:**

Notably uniform parasite populations constituted each farm outbreak, and the parasites from the first and second outbreaks resembled each other to a notable degree, indicating an epidemiological link between them. Wild boar harbored more genetically variable larval cohorts, distinguishing them from parasites isolated from domestic pigs.

**Conclusions:**

Microsatellite markers succeeded in distinguishing isolates of the highly homogeneous *T. spiralis*, aiding efforts to track transmission. Each outbreak was composed of a homogenous group of parasites, suggesting a point source of contamination.

**Graphical abstract:**

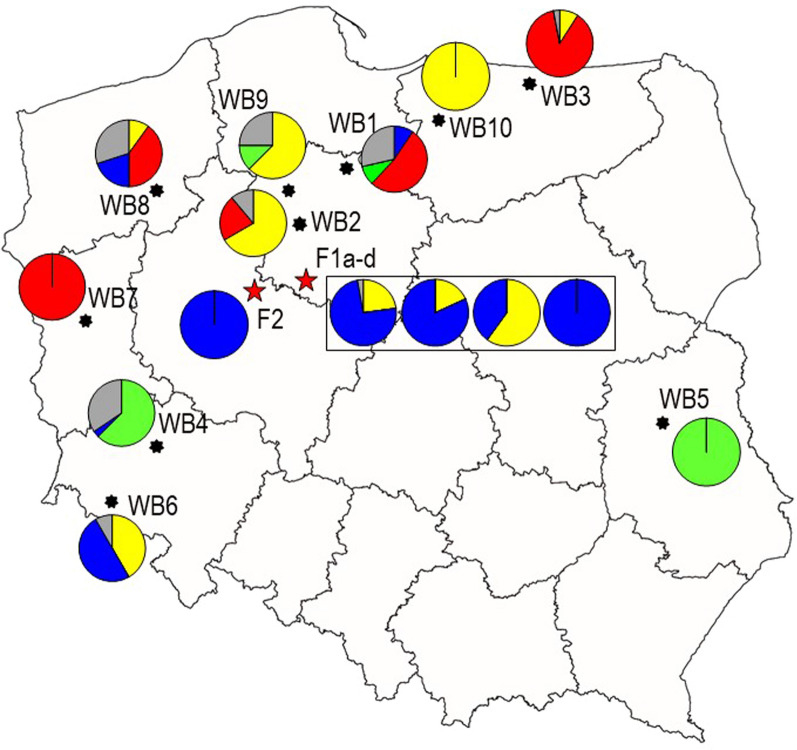

**Supplementary Information:**

The online version contains supplementary material available at 10.1186/s13071-021-04861-9.

## Background

Among parasitic species in the genus *Trichinella, Trichinella spiralis* most frequently causes human infections [1]. The main source of infection is undercooked meat and meat-derived products from pigs and wild boar harboring infective larvae. In the European Union in the last 15 years, prevalent *T. spiralis* infections in domestic animals have been documented in Bulgaria, Poland, Romania, and Spain [2–15]. Each year in Poland, hundreds of wild boar carcasses (6,695 from 2006 to 2019) and dozens of pig carcasses (321 from 2006 to 2019) have been condemned by the Veterinary Inspection Service (VIS) in accordance with EU Reg 2015/1375 [16] governing foodborne *Trichinella* [2–15].

Swine husbandry is especially widespread in the northwestern Polish provinces of Wielkopolskie and Kujawsko-Pomorskie [17]. Here, domesticated pigs reared in backyards may be exposed to *T. spiralis* spillover from wild boar and wild carnivores (when scraps of home-slaughtered pigs and game are disposed of improperly). Synanthropic rats complicate epidemiology further, acting as *T. spiralis* vectors to wild and domesticated animals [18–20]. Historically, it was most common for only one of a farm’s pigs to be diagnosed with infection by the Polish VIS; since 2013, infections in several of a farm’s pigs have been documented; of late, more detailed epidemiological investigations have documented outbreaks involving several to dozens of pigs on a farm. These clustered outbreaks suggest contamination of shared feed, perhaps by means of illegally added meat [21].

Epidemiological investigations clarifying the source of the infection would strengthen countermeasures to lessen livestock exposure and enhance food safety. Veterinary services respond to outbreaks by seeking to improve farm management in ways that limit parasite transmission. At present, although veterinary services determine which species of *Trichinella* caused an outbreak, they are powerless to rule in or rule out specific sources of infection. Tools enabling traceback would aid future efforts to establish sources of infection.

To identify or exclude suspected infection sources, epidemiologists must first understand the extent and distribution of genetic variation in a pathogen population. Previous studies identified notably limited genetic variability in the European *T. spiralis* population. This is true for microsatellite loci [22, 23] as well as for nuclear and mitochondrial genes [22, 24]. Despite this low genetic variability, La Rosa et al. [25] found that among 41 naturally infected wild boars in Extremadura (Spain), many hosts harbored genetically uniform cohorts of larval parasites; others harbored genetically admixed larval cohorts. Although greater genetic variability characterizes European populations of *Trichinella britovi*, isolates show a similar heterogeneous pattern of genetic structure, aiding identification of the origin of a human outbreak in Nice (France) in 2015 [26].

These studies drew inferences from microsatellite genotypes of individual larvae sampled from geographically proximate hosts; they affirmed that detailed analysis of microsatellite genetic variation of isolates might also be useful in investigating outbreaks, two of which occurred in backyard pig farms in Poland in 2013/2014. Therefore, we endeavored to characterize the genetic structure of the parasites causing these outbreaks, determining whether each outbreak was comprised of genetically uniform parasite cohorts, whether parasites causing one outbreak resembled parasites causing the other outbreak, and whether the parasites causing farm outbreaks resembled those circulating in wildlife.

## Material and methods

### Collection of larvae

According to European Commission regulations [16], routine testing for Trichinella spp. is conducted in pigs, wild boars, and other susceptible animals in Poland. The monitoring is provided by accredited field laboratories of Veterinary Inspection Services (VIS), which uses the validated reference magnetic stirrer method [16] for pooled sample digestion to detect *Trichinella* spp. larvae. When *Trichinella* spp.-positive samples are identified by VIS, muscle samples from those positive samples are sent to the National Reference Laboratory for Parasites in Poland (NVRI) for storage in a special biobank and further analyses.

For this study, samples from two farms identified as positive by VIS were targeted for further analysis. Epidemiological investigations were conducted to determine the number of infected animals on the farm, herd management practices, type of feed used, and presence of Trichinella in synanthropic rats. Fresh larvae were extracted at NVRI from both outbreaks in order to investigate the genetic similarity of the parasites on these farms. Trichinella larvae were isolated from three pigs and one brown rat from outbreak 1 and from one pig from outbreak 2. We also used larvae from ten infected wild boars hunted from seven Polish provinces in 2013 and 2014 (Table 1). For each isolate, a sample cohort of 8–35 larvae was analyzed to represent the subpopulation of parasites infecting that individual animal. Therefore, the terms cohort and subpopulation are used interchangeably throughout the article.

**Table 1:** Main features of *Trichinella spiralis* larval cohorts from domesticated (F1a-b and F1d and F2), synanthropic (F1c), and wild animals (WB1-15) investigated by the analysis of microsatellite polymorphisms

Isolate code	N. of tested larvae larvae	Host	Place of origin (province)	ISS code^a^
F1a^b^	35	Pig	Mogilno^b^ (Kujawsko-Pomorskie)	ISS7667
F1b^b^	33	Pig	Mogilno^b^ (Kujawsko-Pomorskie)	ISS7668
F1c^b^	35	Brown rat	Mogilno^b^ (Kujawsko-Pomorskie)	ISS7675
F1d^b^	9	Pig	Mogilno^b^ (Kujawsko-Pomorskie)	ISS7650
F2^c^	11	Pig	Damasławek^c^ (Wielkopolskie)	ISS7659
WB1	21	Wild boar	Kurzbiela (Kujawsko-Pomorskie)	ISS7669
WB2	9	Wild boar	Świecie (Kujawsko-Pomorskie)	ISS7670
WB3	33	Wild boar	Bartoszyce (Warmińsko-Mazurskie)	ISS7672
WB4	32	Wild boar	Lubin (Dolnośląskie)	ISS7673
WB5	10	Wild boar	Włodawa (Lubelskie)	ISS7674
WB6	12	Wild boar	Jarzmanice Zdrój (Dolnośląskie)	ISS7652
WB7	12	Wild boar	Radachów (Lubuskie)	ISS7646
WB8	10	Wild boar	Redło (Zachodnio-Pomorskie)	ISS7661
WB9	8	Wild boar	Gostycyn (Kujawsko-Pomorskie)	ISS7660
WB10	11	Wild boar	Krasin (Warmińsko-Mazurskie)	ISS7656

^a^Number code assigned by the International *Trichinella* Reference Center

^b^Outbreak 1

^c^Outbreak 2

### DNA extraction and molecular identification

Larvae were collected from infected meat by artificial digestion according to the European Commission regulation [16]; DNAs of single larvae were purified using a combination of the Tissue and Hair Extraction Kit (Promega, USA) and the DNA IQ™ System Extraction Kit (Promega, USA). The manufacturer's protocol was scaled to use 20 µl lysis buffer and 80 µl washing volumes for each larva. All sample DNAs were purified in 96-well PCR plates using the robotic 96 BioSprint workstation. DNAs were eluted in 100 µl elution buffer.

*Trichinella spiralis*,* T. britovi*,* Trichinella pseudospiralis* and rarely *Trichinella nativa* have been found in Poland, and the occurrence of mixed infections with different *Trichinella* species is well known [27]. For this reason, DNA samples of single larvae were initially subjected to multiplex PCR [28] for species identification. Larvae that identified as *T. spiralis* were subjected microsatellite genotyping.

### Genotyping

Nine microsatellite markers were amplified using a Veriti™ 96-Well Thermal Cycler (Applied Biosystems, Germany): TS103, TS128, TS130, TS1007, TS1010B, TS1122, TS1131, TS1380, TS1444 [23, 25]. PCR amplifications were performed in 96-well PCR plates sealed with master clean cap strips (Eppendorf, Germany) to reduce tube volume and minimize evaporation. PCR reactions of single larvae were performed in 16 µl using a premixed 2 × PCR Type-it Microsatellite Master Mix (Qiagen, Germany). The PCR solution for each marker contained 8 µl of Master Mix, 6.4 pmol of each primer [22], and 6.5 µl of purified DNA. Each reaction was subjected to 33 PCR cycles as follows: 95 °C for 30 s, 57 °C for 90 s, and 72 °C for 30 s, plus a first step at 95 °C for 5 min and a post elongation step at 60 °C for 30 min. PCR products (i.e. allele) were separated by a Qiaxcel capillary electrophoresis device (Qiagen, Germany) using a high-resolution DNA gel cartridge (Qiagen, Germany). To compare the PCR products a GelPilot 100-bp ladder (Qiagen, Germany) was used as an alignment marker. Results were analyzed by Qiaxcel ScreenGel software version 1.6 (Qiagen, Germany). Alleles were coded for their size in nucleotide base pairs. After each electrophoretic run, some homozygous products were sequenced and used as a reference to define the genotype of all the samples present in the PCR plate [22]. Sequencing of the PCR products was performed using the Sanger method by Macrogen Europe B.V. (Amsterdam, The Netherlands). The sizes of unsequenced alleles (appearing only in heterozygous genotypes) were defined by their electrophoretic migration as compared to sequenced references.

### Genetic variability of larval cohorts

Genetic variability was estimated globally and for each cohort of larvae infecting the same animal. We used FSTAT version 2.9.3 [29] to estimate the number of alleles per locus (*N*_a_), the number of effective alleles (*N*_e_), the observed heterozygosity (*H*_o_), the expected heterozygosity (*H*_e_), and the proportion of polymorphic loci (Pl). The inbreeding coefficient (*F*_is_, Wright index) reported by FSTAT was used to investigate deviations from Hardy-Weinberg equilibrium (HWE) for each sample and globally with significance set at alpha < 0.05. The Bonferroni correction for multiple comparisons was also tested by FSTAT; since no significant value was detected, these results are not shown. The variance of the allele frequencies (*F*_st_) was used to evaluate the similarity of subpopulations in pairwise comparisons [30]. Null allele frequencies were estimated using FreeNA software [31] using the Expectation Maximization (EM) algorithm of Dempster et al. [32]. Principal coordinates analysis (PCoA) in GenAlEx version 6.2 software [33] was used to summarize the *F*_st_ relationships among the larval cohorts. Past version 2.06 software [34] was used to draw the 3D plot from eigenvalues produced by GenAlEx. Populations version 1.2.31 software was used to calculate genetic distances [35] between MLGs of individual larvae and to construct an UPGMA phylogenetic tree of all individuals [36].

### Genetic structure

The genetic relationships among Polish *T. spiralis* larval cohorts were evaluated by the Bayesian clustering algorithm in the software STRUCTURE [37]. The multilocus genotypes (MLG) of 281 muscle larvae, as defined by six polymorphic loci, were submitted to ten independent runs for each of seven scenarios (assigning individuals assuming the existence of *K* population subdivisions where *K* ranged from 2–8). All simulations were performed using the admixture ancestry model, assuming independent frequencies. The run length was set to 500,000 for burn-in period and 1,000,000 for the number of MCMC repetitions. The reliability of cluster patterns obtained for different preassigned K values was evaluated using the method of Evanno et al. [38] as implemented by the Structure Harvester web application [39]. In describing relationships among different hosts, we assigned each larva to a single cluster when its membership probability *Q* was ≥ 0.7. Conversely, admixture was assumed for individuals assigned a membership probability 0.3 < *Q* membership < 0.7.

## Results

### Outbreak backgrounds

One Trichinella outbreak occurred at a pig farm located in Mogilno, Kujawsko-Pomorskie Province (coordinates, lat. 52.7306839-long. 19.0716519), Poland. The farm conducted indoor rearing with a closed breeding cycle. Altogether, 36 pigs were kept in the herd (3 sows and 33 fattening pigs). Pigs were fed with commercial feed supplemented by feed produced by the farmer. In October 2013, routine diagnostic examinations conducted by VIS discovered six pigs infected with Trichinella sp. larvae. Subsequent epidemiological investigation in January 2014 identified 12 additional pigs infected with Trichinella sp. in this herd. Altogether, 18 pigs were infected with *Trichinella* sp. (17 fattening pigs up to 1 year old and 1 4-year-old sow). In addition, 57 brown rats (*Rattus norvegicus*) were trapped on the farm, and Trichinella sp. was discovered in 17 (29.8%) rats. The other outbreak occurred at a pig farm located in Damaslawek, Wielkopolskie Province (coordinates, lat. 52.799003-long. 18.33202), Poland. The farm had 40 pigs in its herd (2 sows, 30 fattening pigs, and 8 piglets). Pigs were housed indoors with a closed breeding cycle and exclusively fed commercial feed. In July 2013, routine testing diagnosed infection in one pig (a sow, 6 years old). Further epidemiological investigations found one other infected pig (a sow, 4 years old) in this herd. None of eight rats captured on this farm were infected with Trichinella.

### Genetic analysis outcome

As detailed below, the cohorts of larvae derived from the outbreaks in each of the two farms bore a remarkable genetic similarity, easily distinguishing them from those collected from wild boar. This similarity was identifiable despite the limited genetic variability in the parasite population as a whole.

### Genetic variability

All 281 larvae from 15 animals were identified as *T. spiralis* by multiplex PCR. Three of nine microsatellites (TS130, TS1131 and TS1444) were entirely fixed for a single allele in all larvae. These were excluded from ensuing analyses because they provided no information on population subdivision. No host harbored larvae characterized by private alleles. The remaining six microsatellite markers (TS103, TS128, TS1007, TS1010B, TS1122, and TS1380) were polymorphic. An average of 18.60 (SD = 1.15) larvae per host produced a reliable and reproducible amplification pattern; of 1,686 amplifications, only 1% produced negative or inconclusive genotypes (0.4% in TS103 and TS1122; 1.8% in TS128; 2% in TS1380) (see Additional file 1). Larvae from the WB1 wild boar were polymorphic at all six loci (Pl = 100%). By contrast, larvae from the F1d and F2 pigs originating from the two farms and larvae from the WB10 wild boar were fixed for a single allele at all six loci (Pl = 0%). The polymorphism of the larvae of the other hosts ranged from 16 to 50% (Table 2). Analysis using FreeNA indicated that null alleles occurred at low enough frequencies to disregard them as sources of analytic bias. We therefore performed subsequent analysis without concern for inflated estimates of homozygosity. Global analyses identified only limited genetic variability, as estimated by *N*_a_ (1.42, SE = 0.07), *N*_e_ (1.18, SE = 0.04), *H*_o_ (0.10, SE = 0.02), and *H*_e_ (0.11, SE = 0.02) (Table 2). Larval genotypes were not distributed randomly among hosts, and global analysis of genotypic frequencies departed significantly from Hardy-Weinberg equilibrium expectations at each marker (confidence limit of *P* set to 0.05).

**Table 2:** Summary of the genetic variability in 15 *Trichinella spiralis* larval cohorts. F1a-d samples from outbreak 1, F2 from outbreak 2, and WB1-10 from hunted wild boar

Pop		*N*	*N*_a_	*N*_e_	*H*_o_	*H*_e_	Pl (%)	*F*_is_
F1a								
Mean		34.8	1.5	1.1	0.04	0.05	50.00	0.213
SE		0.2	0.2	0.1	0.03	0.04		
F1b								
Mean		32.8	1.3	1.0	0.03	0.04	33.33	0.186
SE		0.2	0.2	0.0	0.02	0.03		
F1c								
Mean		34.7	1.2	1.1	0.10	0.07	16.67	− 0.417^a^
SE		0.2	0.2	0.1	0.10	0.07		
F1d								
Mean		9.0	1.0	1.0	0.00	0.00	0.00	NA
SE		0.0	0.0	0.0	0.00	0.00		
F2								
Mean		11.0	1.0	1.0	0.00	0.00	0.00	NA
SE		0.0	0.0	0.0	0.00	0.00		
WB1								
Mean		20.7	2.7	1.6	0.31	0.33	100.00	0.093
SE		0.2	0.5	0.2	0.07	0.07		
WB2								
Mean		9.0	1.7	1.1	0.11	0.10	50.00	− 0.055
SE		0.0	0.3	0.1	0.05	0.04		
WB3								
Mean		32.5	1.5	1.3	0.17	0.17	50.00	− 0.01
SE		0.3	0.2	0.1	0.09	0.08		
WB4								
Mean		31.8	1.5	1.3	0.13	0.15	50.00	0.182
SE		0.2	0.2	0.2	0.07	0.09		
WB5								
Mean		9.8	1.2	1.1	0.04	0.06	16.67	0.407
SE		0.2	0.2	0.1	0.04	0.06		
WB6								
Mean		12.0	1.5	1.2	0.07	0.10	50.00	0.375
SE		0.0	0.2	0.1	0.04	0.07		
WB7								
Mean		12.0	1.5	1.4	0.14	0.18	33.33	0.262
SE		0.0	0.3	0.2	0.09	0.11		
WB8								
Mean		9.8	1.5	1.5	0.22	0.24	50.00	0.114
SE		0.2	0.2	0.2	0.12	0.11		
WB9								
Mean		8.0	1.3	1.2	0.10	0.11	33.33	0.146
SE		0.0	0.2	0.1	0.07	0.07		
WB10								
Mean		11.0	1.0	1.0	0.00	0.00	0.00	NA
SE		0.0	0.0	0.0	0.00	0.00		
Total								
Mean		18.60	1.42	1.18	0.10	0.11	0.36	
SE		1.15	0.07	0.04	0.02	0.02	0.07	

*N*, average number of individuals per locus; *N*_a_, average number of alleles per locus; *N*_e_, average effective number of alleles per locus; *H*_o_: average observed heterozygosity per locus, *H*_e_: average expected heterozygosity per locus; Pl: percentage of polymorphic loci; *F*_is_, inbreeding coefficient; SE, standard error; NA, not applicable

^a^Significant deviation from Hardy-Weinberg expectation (nominal *P* level set to 0.05)

*F*_is_ values estimated from genotypic frequencies of six variable markers for each sample identified a significant deviation from Hardy-Weinberg equilibrium only in the cohort of the brown rat (isolate code F1c) (i.e. it showed a significantly smaller *F*_is_ than observed; *P* = 0.011) (Table 2).

### Genetic comparison between larval cohorts

To identify similarities or differences among parasites from different hosts, *F*_st_ was estimated for all pairs of larval cohorts (Table 3). *F*_st_ ranged from 0 to 1. Almost all such comparisons (92.4%) significantly distinguish cohort pairs (Table 3). The average pairwise *F*_st_ estimate was 0.474, indicating substantial differences between most larval cohorts. Two comparisons reached maximal differentiation (*F*_st_ = 1): wild boar WB10 *vs* rat F1d or *vs* pig F2. Notable exceptions occurred when comparing larvae from hosts derived from the two outbreaks; *F*_st_ = 0 when comparing larvae of pig F1a to pig F1b from outbreak 1 and when comparing rat F1d from outbreak 1 to pig F2 from outbreak 2. Notably, the *F*_st_ variation attributable to hosts involved in domestic outbreaks (average *F*_st_ = 0.070, 95% CI 0.016– 0.124) was significantly lower than the pairwise comparisons of larvae infecting wild hosts (*F*_st_ = 0.417, 95% CI 0.344–0.490); *t* = 4,449 and *P* << 0.001 (Table 3; see Additional file 2).

**Table 3:** *F*_st_ pairwise comparisons among 15 *Trichinella spiralis* larval cohorts

Isolate code	F1a	F1b	F1c	F1d	F2	WB1	WB2	WB3	WB4	WB5	WB6	WB7	WB8	WB9
F1b	−**0.006**													
F1c	0.041	0.099												
F1d	**0.050**	**0.020**	0.197											
F2	**0.060**	**0.028**	0.207	**0.000**										
WB1	0.462	0.503	0.385	0.422	0.442									
WB2	0.604	0.700	0.452	0.762	0.785	0.045								
WB3	0.626	0.665	0.548	0.629	0.645	0.047	0.107							
WB4	0.549	0.582	0.492	0.544	0.557	0.391	0.474	0.549						
WB5	0.907	0.932	0.879	0.952	0.957	0.518	0.838	0.688	0.695					
WB6	**0.081**	0.164	–**0.005**	0.235	0.261	0.249	0.296	0.450	0.390	0.843				
WB7	0.753	0.788	0.716	0.730	0.747	0.303	0.506	0.383	0.673	0.776	0.624			
WB8	0.420	0.486	0.337	0.407	0.434	0.102	0.164	0.206	0.436	0.718	0.202	0.308		
WB9	0.681	0.764	0.557	0.819	0.840	0.135	0.124	0.307	0.310	0.812	0.374	0.631	0.271	
WB10	0.727	0.821	0.588	1.000	1.000	0.178	0.076	0.310	0.525	0.934	0.489	0.720	0.354	0.237

F1a-d isolates collected from outbreak 1, F2 from outbreak 2 and WB1-15 from hunted wild boars. Comparisons in bold are not significantly different from null hypothesis (nominal *P* level set to 0.05)

**Fig. 1:**
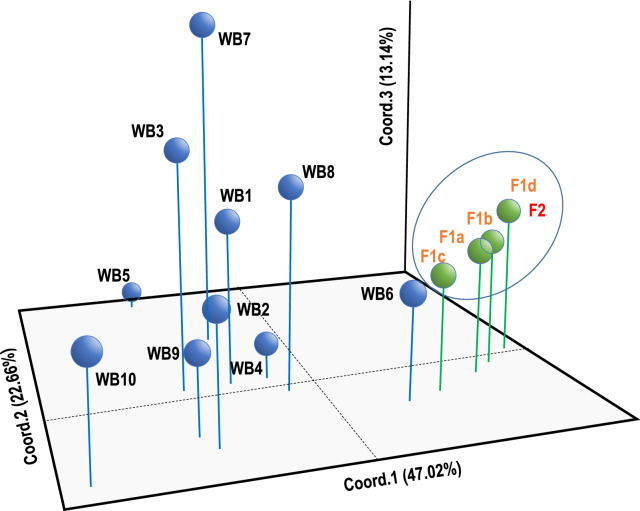
Principal Coordinates Analysis (PCoA) of 15 Polish larval cohorts. Three-dimensional plot showing the spatial relationships among samples studied based on *F*_st_ pairwise value matrix. The percentages of variance explained by the three main components are given on axes (47.02, 22.66, and 13.14, respectively). Green spheres refer to larval cohorts from outbreak 1 (orange font) and outbreak 2 (red font); black spheres refer to larval cohorts collected from wild boar. Circles are drawn to emphasize groupings of isolates from outbreaks 1 and 2

The differentiation among the *F*_st_ values displayed in Table 3 is visually summarized by multivariate principal coordinates analysis (Fig. 1) that attributes, by the three main axes, > 82% of the variance of the eigenvalues. Figure 1 shows that larval cohorts belonging to the two outbreaks are distinct from all the other larval cohorts, except for larvae from the WB6 wild boar (which appears proximate to the two outbreak cohorts).

**Fig. 2:**
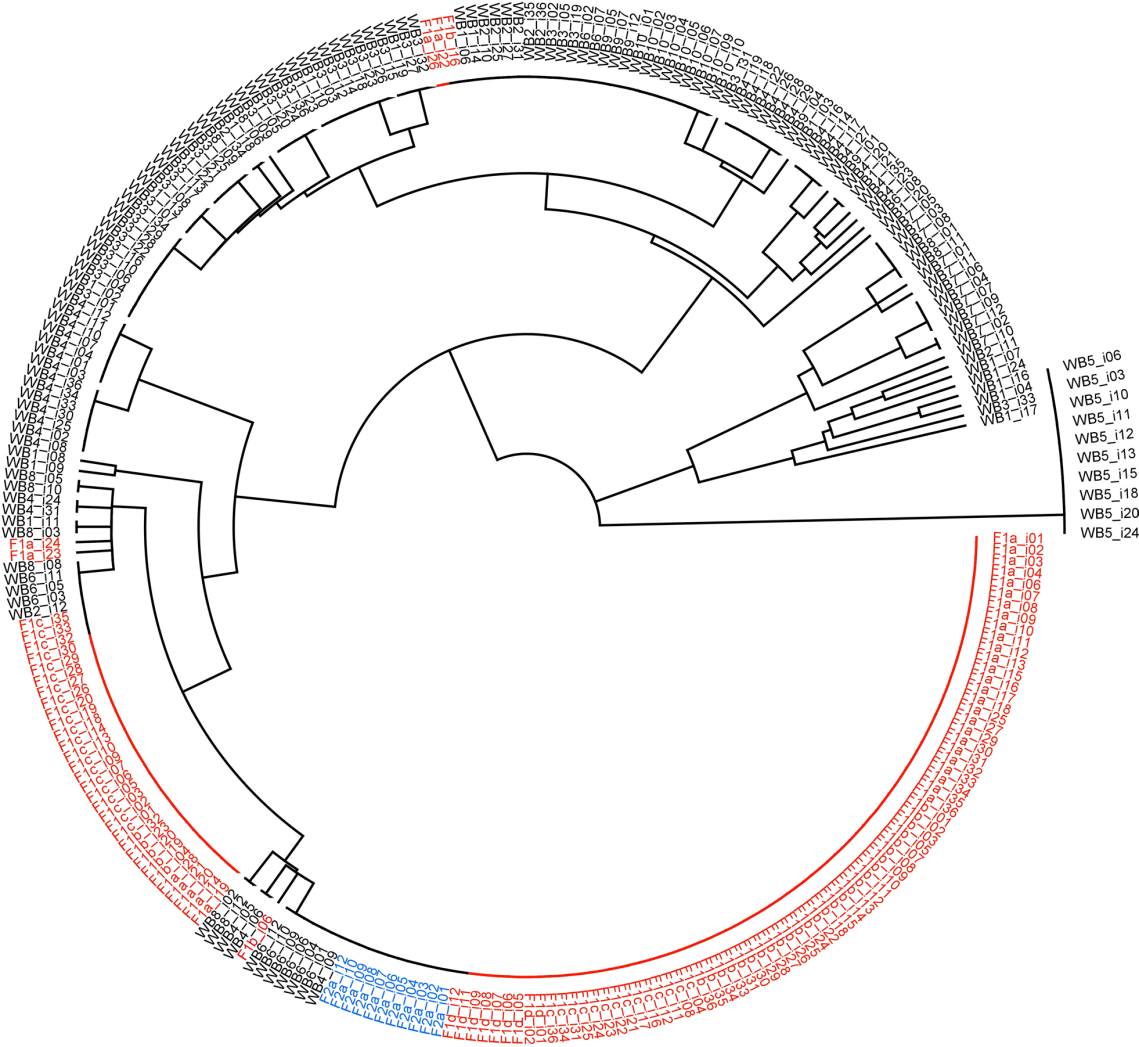
UPGMA phylogenetic tree of all individual *Trichinella spiralis* larvae based on genetic distances between their multilocus genotypes. Individuals from outbreak 1, outbreak 2, and wild boar are signed in red, blue and black, respectively. Codes as in Table 1

The UPGMA phylogenetic tree constructed from Nei et al. [36] genetic distances between MLGs of individuals illustrates relationships among them, again highlighting the resemblance of parasites causing the two farm outbreaks. The neighbor-joining algorithm placed all but three larvae from the farm outbreaks into either of two adjacent, minimally differentiated subclades (Fig. 2). In these clades, 21 individuals from wild boar were also located [WB6 (*n* = 9), WB8 (*n* = 6), WB4 (*n* = 5), and WB2 (*n* = 1)]. The remaining larvae isolated from wild boar were more distant in the tree and variable among hosts.

### Genetic structure

A Bayesian algorithm implemented by STRUCTURE explored genetic subdivision among sampled individuals. The STRUCTURE simulations (subdividing the total into *k* = 2–8) affirmed that outbreak cohorts resembled each other more than they resembled isolates from wild boar (see Additional file 3) and that each wild boar cohort differed markedly from other each other.

**Fig. 3:**
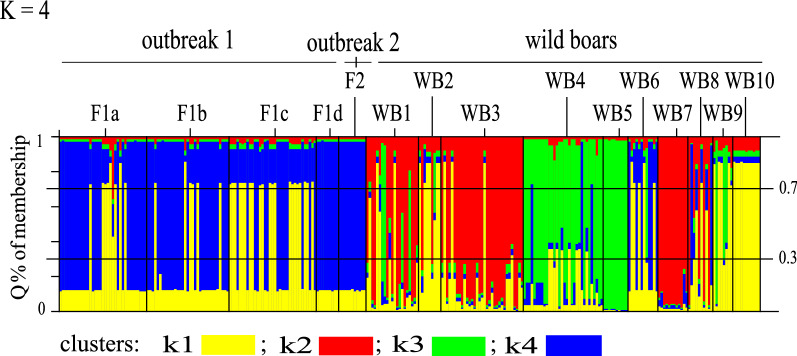
Bayesian analysis of the multilocus genotypes of individuals from 15 larval cohorts. The plot shows the result of the simulation conducted by STRUCTURE using a value of *k* = 4, discussed in the text as best describing the dataset. Individual larva are represented by a single vertical line divided into different colored segments based on the estimates of belonging to each cluster predicted for that simulation; the *Q* values of 0.3 and 0.7 are marked. Larval cohorts F1a-d from outbreak 1, F2 from outbreak 2, and WB1-10 from wild boar

**Fig. 4:**
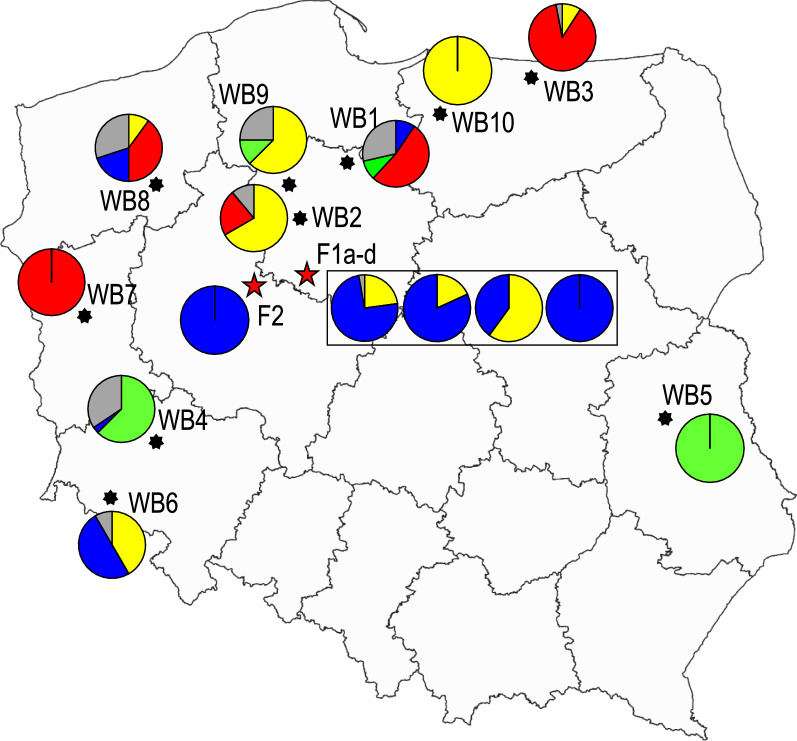
Geographic distribution of genetic clusters in Poland as defined by *K* = 4 STRUCTURE simulation. Each circle represents a given cohort as in Fig. 3 (*k* = 4 simulation). Each section shows the percentage of larvae assigned to a specific cluster by a *Q* value ≥ 0.70; the grey sector represents the percentage of admixed individuals. Red stars represent the two pig outbreaks. The rectangle encloses the cohorts collected from outbreak 1

Following the criteria established by Evanno et al. [38] to estimate the degree of subdivision most consistent with available data, the weight of evidence here suggests approximately three or four population subdivisions (see Additional file 4). However, assuming four subdivisions enabled the largest number of individuals (91%) to be assigned to a given population with *Q* probability values ≥ 0.7 (simulation is shown in Fig. 3). (For completeness, we illustrate the results of simulations from *k* = 2 to *k* = 5 in Additional file 5, panel A and B; see also Additional file 6). Figure 4 summarizes the genetic structure of the 15 cohorts, as defined by *k* = 4 simulations, according to their geographical origin. Each circle represents a given larval cohort and each section shows the proportion of individual larvae assigned to a specific cluster by a *Q* value ≥ 0.70 (see Additional file 5, panel A). The percentage of individuals determined to be admixed (when the *Q* value did not reach 0.70 for any of the 4 clusters) is marked in grey.

The following were observed in the *k* = 4 simulations. This is consistent with STRUCTURE analyses using other values of k with the entire dataset (Fig. 3 and Additional file 3).

- STRUCTURE often assigned each of a host’s larvae to the same cluster with > 70% certainty (*Q* ≥ 0.7) (suggesting these larvae were highly inbred). This occurs for the F1d pig of farm 1, the F2 pig of farm 2, and the WB5, Wb7, and WB10 wild boars. These samples had low allelic heterogeneity, resulting in minimal genetic structure. All individuals from pigs F1d and F2 were assigned to the same cluster, suggesting that these infections share the same recent ancestors, while individuals from WB5, WB7, and WB10, were assigned to different clusters, and separate ancestors are suggested.
- Larval cohorts from wild boars WB1, WB2, WB4, WB8, and WB9 included many larvae assigned with intermediate Q probability to more than one (of 4) hypothesized subdivisions (0.3 < *Q* membership < 0.7). A mixture of at least two lineages may have recently contributed to the ancestry of these larvae. Larva F1a/22 was unique among samples F1a-c in showing admixture with Q % of membership = 0.44 for cluster-1 and *Q* % of membership = 0.35 for cluster-2 (Fig. 3 and Additional file 5, panel B) prompting further investigation.
- The larval cohorts of pigs F1a and F1b and rat F1c from farm 1 and WB3 and WB6 wild boars each included individuals assigned to two different clusters (*Q* ≥ 0.7) with little to no admixture between them. In cohorts F1a–c, mixed infection sources could be responsible for the patterns of cluster membership; they were analyzed independently with STRUCTURE, as in La Rosa et al. [25], but no subdivision into separate clusters was established.

## Discussion

We investigated *Trichinella* sp*.* outbreaks on pig farms in Poland in 2013 and 2014, identifying infected pigs and rats. The prior epidemiological investigation of outbreak 1 substantiated the suspicion that contaminated meat may have been the source of infection [40]. Here, genetic data affirm the likelihood that a single infection source gave rise to all infections (documented in half of the herd and in 17 synanthropic rats). In the second outbreak, a source of infection had not previously been established. However, the epidemiological inquiry indicated that illegal movements of pigs between these farms had probably occurred. Molecular tests previously identified *T. spiralis* as the parasite involved in both outbreaks, but it was not possible to define whether the two outbreaks had the same origin.

Attempts to distinguish among European isolates of *T. spiralis* have been limited by low genetic variability [24, 41]. Recently, the development of new molecular tools enabled characterization of the genetic structure of individual larvae of the same species, making distinction of genetic differences between two isolates possible [25, 26]. Here, we confirmed for the first time that two *T. spiralis* isolates, from two separate outbreaks, were notably more similar to each other than would be expected for randomly chosen larval cohorts, supporting the hypothesis that they were somehow related. Despite the limited genetic variability shown by *T. spiralis* in Europe, the suspicion of a link between the two outbreaks 40 km apart was confirmed by demonstrable genetic affinities (from pigs and a rat).

We reached this conclusion despite low overall genetic variability, consistent with prior regional estimates. Across all investigated loci, we observed globally an average *N*_a_ of only 1.42 alleles/locus and an average *H*_o_ of 0.10. These low values are comparable to prior estimates from 41 wild boar larval cohorts (genotyped using 5 polymorphic loci) from Extremadura (Spain) (*N*_a_ = 1.59 and *H*_o_ = 0.17) [25] and from 13 cohorts (using 7 loci) collected from throughout Europe (*N*_a_ = 1.27 and *H*_o_ 0 = 0.09) [23]. By contrast, Asian *T. spiralis* cohorts harbor more genetic variability [22, 23]. Recently, Li et al. [42] recognized 7 to 19 alleles/locus studying 16 new microsatellite markers of 12 Chinese isolates. Why Asia and Europe differ so markedly in their polymorphism remains to be determined, but the practical effect has been to severely limit the application of molecular epidemiology to discriminate among *T. spiralis* isolates in Europe. These tools have adjudicated epidemiological hypotheses for *T. britovi*, which harbors more variation and therefore more discernable geographic structure [26]. Asia may have been a source of European *T. spiralis* spread or may simply have maintained larger effective populations for longer [22, 43].

Previous studies have shown that individual hosts generally harbor larval cohorts that can be distinguished, to greater or lesser extents, from larval cohorts of other individual hosts [23]. Here, pairwise *F*_st_ among larval cohorts from ten wild boars ranged from 0.045 to 0.934 (average 0.52). The seemingly large differences between cohorts reflects the biology of cohort formation: each host ingests a small number of infectious larvae that then have limited opportunities to outcross because the larvae from a particular meal reach adulthood at the same time. The result is frequent inbreeding, which minimizes the genetic variability of each the larval cohort in any host. Only rarely will a host eat sequential independent *T. spiralis*-infected meals, producing genetically distinct adults maturing at the same time in the same gut, enabling outcrossing. These rare occurrences can increase genetic diversity locally and limit allelic loss through genetic drift. From a molecular epidemiological perspective, these differences provide opportunities to distinguish infections, even when allelic diversity is limited.

Prior epidemiological investigations of these outbreaks raised suspicion that pigs had been moved between the investigated farms; one farm likely introduced *T. spiralis* to the other. This study sustains this suggestion by identifying notably little genetic differentiation among the larvae derived from the five hosts on the two farms studied. The larvae sampled on farms account for 7% of the total genetic variation (mean *F*_st_ = 0.07, 95% CI 0–0.21). When wild boars were considered, six times as much genetic variation was observed (mean *F*_st_ = 0.417), indicating that the larvae of outbreak cohorts share notably recent common ancestors.

The multivariate analysis, based on the *F*_st_ matrix, confirms the genetic similarity of the larvae collected from the two outbreaks. The five larval cohorts from the four domestic pigs and from the rat trapped at the pig farm define a cluster distinct from all the larva cohorts from all sampled wild boar except for wild boar WB6; as shown in Fig. 1, nine out of ten larval cohorts from wild boar are scattered in the 3D space, supporting their genetic independence.

The UPGMA phylogenetic tree of MLGs of all individual larvae divided almost all individuals from wild boar from individuals isolated from outbreak hosts, creating a ‘domestic’ subclade within a ‘wild’ clade (Fig. 2). The ‘wild’ clade is characterized by larger genetic distances between MLGs, suggesting the circulation of a wider subset of genotypes among wildlife. The ‘domestic’ subclade includes mostly uniform genotypes from domestic pigs and the synanthropic rat, larvae from the WB6 wild boar, and several individual larvae from other wild boar, suggesting that the genotypes of the domestic infections are likely a subset of wildlife genotypes. In addition, these data suggest that the four animals sampled on farm 1 harbored larvae from a single source. These results support the preliminary suspicion that contaminated feed on farm 1 was likely a shared source of infection for the half of the pig herd and rats.

Analyzing multilocus microsatellite genotypes using the Bayesian algorithm in STRUCTURE confirmed epidemiological assessment despite the limited variability characterizing these isolates. This is similar to what has previously been reported in *T. spiralis* in Spain and *T. britovi* on two Mediterranean islands [23, 26]. Isolates derived from populations generally lacking in genetic variability (as is true for *T. spiralis* in Europe) may become distinct through successive passages, since inbred parasites lose alleles through genetic drift. Here, some larval cohorts are comprised of individuals assigned to a single cluster (each individual has *Q* > 0.7) but differing from one another (i.e. WB5, WB7, WB10, and domestic isolates F1d and F2). This suggests that these cohorts likely have two lineages that have undergone generations of inbreeding. Other cohorts harbor admixed individuals showing a plethora of different patterns of membership (0.3 < *Q* > 0.7), indicating that they may have undergone more recent admixture. This pattern occurred primarily in cohorts from wild boar, indicating significant gene flow among wildlife isolates. More hosts likely contribute to the sylvatic cycle, since wild hosts will eat from multiple infected carcasses over their lifetime. The admixed patterns in the wild samples described here likely reflect the history of sequential infections in the lifespan of wild animals.

However, the evolutionary forces driving genetic diversity depend on the amount of genetic variability in a population. In cases of species as homogeneous as *T. spiralis* in Europe, which has a small effective population size, the admixture level is limited by the number of genotypes circulating in the environment [44]. Of the parasites sampled from wild boar, those of WB6 most closely resembled those from the two farms; this is notable, given how far away WB6 was hunted from these farms. Low overall genetic variability of *T. spiralis* in Poland increases the likelihood that geographically disparate isolates share genetic similarities.

In *Trichinella* spp., transmission of larvae from wild to domestic hosts typically occurs as a single, isolated event. The limited number and relatedness of larvae ingested in a single meal often results in further inbreeding in the domestic host. This inbreeding leads to clonal expansions that differ from subpopulations in other hosts. We have demonstrated that epidemiologically related infections can be recognized as especially similar even when genetic variability is low (with fewer than two alleles per locus and an expected heterozygosity of only 10%). This was only achieved through the use of microsatellite loci, which proved variable, despite the low genetic variability among European *T. spiralis* subpopulations. Although outbreak tracing would be further aided by greater polymorphism or the occurrence of private alleles, we conclude that these markers can help traceback efforts even under conditions of limited overall genetic variability.

## Conclusions

This study shows that, despite the low genetic variability found in the *T. spiralis* population, microsatellite genotyping allows for the differentiation of the outbreak isolates from randomly selected wild boar. The clonal character of isolates from farm 1 confirms the preliminary diagnosis that these animals contracted infection from a shared source (most likely feed containing infecting *Trichinella* larvae). Moreover, we confirm the suspicion of a link between the two investigated farms, finding high genotypic similarity of larval cohorts within both farms. These conclusions were achieved using microsatellites markers capable of surmounting limited genetic variability. Other methods of sampling genetic or genomic variation may similarly contribute to defining transmission routes, identifying sources, and preventing the spread of infection. Methods capable of resolving such distinctions more quickly would have even greater application to real-time source tracking.

## Supplementary Information

**Additional file 1. **Allele frequencies and sample sizes. N = number of tested larvae per 15 larval cohorts of *Trichinella spiralis* from domesticated (F1a, F1b, F1d and F2), synanthropic (F1c) and wild animals (WB1-10).

**Additional file 2. **Student's *t*-test for equal means. Comparison of F_st_ values between outbreak larval cohorts *vs* F_st_ values between wild boar larval cohorts. Bootstrap N = 9999, permutations N = 9999.

**Additional file 3. **Bayesian analysis of the multilocus genotypes of 281 individuals from 15 larval cohorts.

**Additional file 4. **Evaluation of the optimal number of genetic clusters (k) present in Polish *Trichinella spiralis* subpopulations applying method proposed by Evanno et al. [38].

**Additional file 5. **Inferred Q percentage of membership of each individual for different values of k.

**Additional file 6. **When no *a priori* degree of subdivision can be assumed for a population, STRUCTURE affords the means to consider how biological samples cohere, genetically, assuming greater or lesser degrees of subdivision.

## Data Availability

The data supporting the conclusions of this article are included within the article and Additional files. Raw data are available at the Istituto Superiore di Sanità (Dr. Giuseppe La Rosa, email: giuseppe.larosa@iss.it).

## Abbreviations

VIS: Veterinary Inspection Service
*N*_a_: Mean number of alleles per locus
*N*_e_: Effective number of alleles
*H*_o_: Observed heterozygosity
*H*_e_: Expected heterozygosity
Pl: Proportion of polymorphic loci
HWE: Hardy-Weinberg equilibrium
*F*_is_: Wright’s inbreeding coefficient
*F*_st_: Wright’s fixation index
MLG: Multilocus genotype
PCoA: Principal coordinates analysis
*Q*: Estimated membership coefficients
