# Supplementary material for: Genetic evidence substantiates transmission of Trichinella spiralis from one swine farm to another
Source: Parasit Vectors. 2021 Jul 9;14:359. doi: 10.1186/s13071-021-04861-9 (PMC8268521; doi:10.1186/s13071-021-04861-9)
Supplement: Supplementary file 1 — Additional file 1. Allele frequencies and sample sizes. N = number of tested larvae per 15 larval cohorts of Trichinella spiralis from domesticated (F1a, F1b, F1d and F2), synanthropic (F1c) and wild animals (WB1-10). [file 13071_2021_4861_MOESM1_ESM.docx]

Additional file 1 - Allele frequencies and sample sizes. N = number of tested larvae per 15 larval cohorts of *Trichinella spiralis* from domesticated (F1a, F1b, F1d and F2), synanthropic (F1c) and wild animals (WB1-10).

|  |  |  | Outbreak 1 | | Outbreak 2 | | |  |  |  | Wild boars | |  |  |  |  |
| --- | --- | --- | --- | --- | --- | --- | --- | --- | --- | --- | --- | --- | --- | --- | --- | --- |
| Locus | Allele^a^ | F1a | F1b | F1c | F1d | F2 | WB1 | WB2 | WB3 | WB4 | WB5 | WB6 | WB7 | WB8 | WB9 | WB10 |
| TS103 | N | 35 | 33 | 35 | 9 | 11 | 21 | 9 | 33 | 32 | 10 | 12 | 12 | 9 | 8 | 11 |
|  | 161 | 0.16 | 0.11 | 0.30 | 0.00 | 0.00 | 0.86 | 0.89 | 1.00 | 0.53 | 1.00 | 0.38 | 0.54 | 0.56 | 1.00 | 1.00 |
|  | 169 | 0.84 | 0.89 | 0.70 | 1.00 | 1.00 | 0.14 | 0.11 | 0.00 | 0.47 | 0.00 | 0.62 | 0.46 | 0.44 | 0.00 | 0.00 |
| TS128 | N | 35 | 33 | 34 | 9 | 11 | 21 | 9 | 31 | 31 | 9 | 12 | 12 | 10 | 8 | 11 |
|  | 212 | 1.00 | 1.00 | 1.00 | 1.00 | 1.00 | 0.57 | 0.89 | 0.39 | 0.97 | 0.22 | 1.00 | 0.00 | 0.60 | 1.00 | 1.00 |
|  | 216 | 0.00 | 0.00 | 0.00 | 0.00 | 0.00 | 0.43 | 0.11 | 0.61 | 0.03 | 0.78 | 0.00 | 1.00 | 0.40 | 0.00 | 0.00 |
| TS1007 | N | 35 | 33 | 35 | 9 | 11 | 21 | 9 | 33 | 32 | 10 | 12 | 12 | 10 | 8 | 11 |
|  | 174 | 1.00 | 1.00 | 1.00 | 1.00 | 1.00 | 0.90 | 1.00 | 1.00 | 0.22 | 0.00 | 0.96 | 1.00 | 1.00 | 0.75 | 1.00 |
|  | 177 | 0.00 | 0.00 | 0.00 | 0.00 | 0.00 | 0.10 | 0.00 | 0.00 | 0.78 | 1.00 | 0.04 | 0.00 | 0.00 | 0.25 | 0.00 |
| TS1010B | N | 35 | 33 | 35 | 9 | 11 | 21 | 9 | 33 | 32 | 10 | 12 | 12 | 10 | 8 | 11 |
|  | 244 | 0.01 | 0.02 | 0.00 | 0.00 | 0.00 | 0.02 | 0.00 | 0.00 | 0.00 | 0.00 | 0.00 | 0.00 | 0.00 | 0.00 | 0.00 |
|  | 246 | 0.00 | 0.00 | 0.00 | 0.00 | 0.00 | 0.07 | 0.00 | 0.00 | 0.00 | 0.00 | 0.04 | 0.00 | 0.00 | 0.00 | 0.00 |
|  | 248 | 0.00 | 0.00 | 0.00 | 0.00 | 0.00 | 0.07 | 0.06 | 0.00 | 0.00 | 1.00 | 0.00 | 0.00 | 0.00 | 0.00 | 0.00 |
|  | 250 | 0.99 | 0.98 | 1.00 | 1.00 | 1.00 | 0.62 | 0.88 | 0.86 | 1.00 | 0.00 | 0.96 | 1.00 | 1.00 | 1.00 | 1.00 |
|  | 252 | 0.00 | 0.00 | 0.00 | 0.00 | 0.00 | 0.22 | 0.06 | 0.14 | 0.00 | 0.00 | 0.00 | 0.00 | 0.00 | 0.00 | 0.00 |
| TS1122 | N | 35 | 33 | 35 | 9 | 11 | 20 | 9 | 33 | 32 | 10 | 12 | 12 | 10 | 8 | 11 |
|  | 169 | 0.00 | 0.00 | 0.00 | 0.00 | 0.00 | 0.08 | 0.00 | 0.00 | 0.00 | 0.00 | 0.00 | 0.00 | 0.00 | 0.19 | 0.00 |
|  | 181 | 1.00 | 1.00 | 1.00 | 1.00 | 1.00 | 0.92 | 1.00 | 1.00 | 1.00 | 1.00 | 1.00 | 1.00 | 1.00 | 0.81 | 1.00 |
| TS1380 | N | 34 | 32 | 34 | 9 | 11 | 20 | 9 | 32 | 32 | 10 | 12 | 12 | 10 | 8 | 11 |
|  | 252 | 0.00 | 0.00 | 0.00 | 0.00 | 0.00 | 0.03 | 0.00 | 0.00 | 0.00 | 0.00 | 0.00 | 0.21 | 0.35 | 0.00 | 0.00 |
|  | 255 | 0.02 | 0.00 | 0.00 | 0.00 | 0.00 | 0.20 | 0.00 | 0.19 | 0.00 | 0.00 | 0.00 | 0.58 | 0.00 | 0.00 | 0.00 |
|  | 267 | 0.98 | 1.00 | 1.00 | 1.00 | 1.00 | 0.77 | 1.00 | 0.81 | 1.00 | 1.00 | 1.00 | 0.21 | 0.65 | 1.00 | 1.00 |

^a^ alleles are coded according to their size in nucleotide number.
