# Supplementary material for: Genetic evidence substantiates transmission of Trichinella spiralis from one swine farm to another
Source: Parasit Vectors. 2021 Jul 9;14:359. doi: 10.1186/s13071-021-04861-9 (PMC8268521; doi:10.1186/s13071-021-04861-9)
Supplement: Supplementary file 2 — Additional file 2. Student's t-test for equal means. Comparison of Fst values between outbreak larval cohorts vs Fst values between wild boar larval cohorts. Bootstrap N = 9999, permutations N = 9999. [file 13071_2021_4861_MOESM2_ESM.docx]

Additional file 2 - Student's t-test for equal means. Comparison of F_st_ values between outbreak larval cohorts *vs* F_st_ values between wild boar larval cohorts. Bootstrap N = 9999, permutations N = 9999.

|  | Wild boar larval cohorts | Outbreaks 1 and 2 larval cohorts |
| --- | --- | --- |
| N | 45 | 10 |
| Mean | 0.41707 | 0.0702 |
| 95% conf | 0.34426-0.48988 | 0.016274-0.12413 |
| Variance | 0.058734 | 0.0056826 |
| t | 4.4494 |  |
| p (same mean) | 4.45E-05 |  |
| Critical t value (*P* = 0.05) | 2.0057 |  |
