# Supplementary material for: Genetic evidence substantiates transmission of Trichinella spiralis from one swine farm to another
Source: Parasit Vectors. 2021 Jul 9;14:359. doi: 10.1186/s13071-021-04861-9 (PMC8268521; doi:10.1186/s13071-021-04861-9)

Additional file 3 - Bayesian analysis of the multilocus genotypes of 281 individuals from 15 larval cohorts. The plots shows the simulations conducted by STRUCTURE increasing k from 2 to 8. Each color in any simulation is representative of a genetically distinct cluster. Individual larva are represented by a single vertical line divided into different colored segments based on the estimates of belonging to each cluster predicted for that simulation. The simulation performed using k = 4 and discussed in the text as best describing the dataset is boxed; the Q values of 0.3 and 0.7 are marked. Larval cohorts F1a-d from outbreak 1, F2 from outbreak 2 and WB1-10 from wild boar.

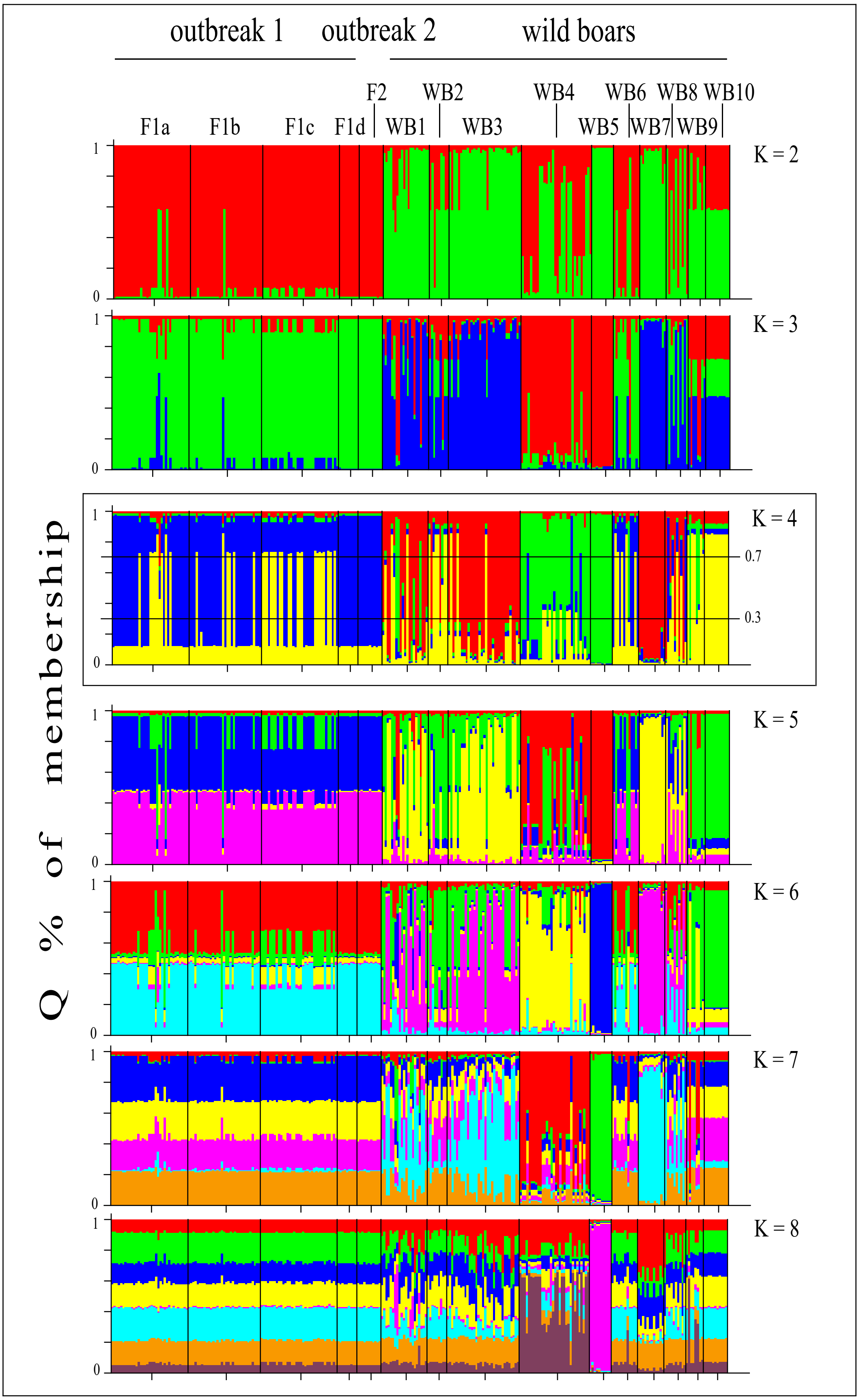

Supplement: Supplementary file 3 — Additional file 3. Bayesian analysis of the multilocus genotypes of 281 individuals from 15 larval cohorts. [file 13071_2021_4861_MOESM3_ESM.pdf]
