# Supplementary material for: Genetic evidence substantiates transmission of Trichinella spiralis from one swine farm to another
Source: Parasit Vectors. 2021 Jul 9;14:359. doi: 10.1186/s13071-021-04861-9 (PMC8268521; doi:10.1186/s13071-021-04861-9)

Additional file 4 - Evaluation of the optimal number of genetic clusters (k) present in Polish *Trichinella spiralis* subpopulations applying method proposed by Evanno et al. [38].

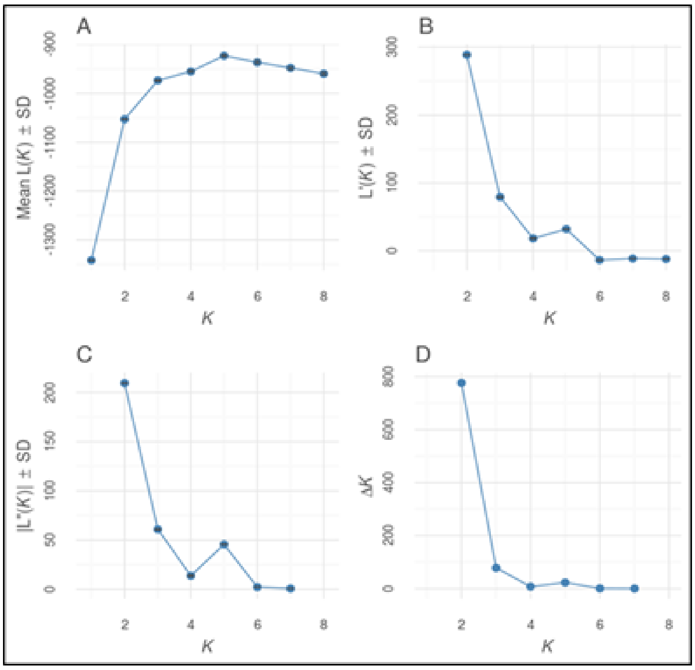

Supplement: Supplementary file 4 — Additional file 4. Evaluation of the optimal number of genetic clusters (k) present in Polish Trichinella spiralis subpopulations applying method proposed by Evanno et al. [38]. [file 13071_2021_4861_MOESM4_ESM.pdf]
