# Supplementary material for: Genetic evidence substantiates transmission of Trichinella spiralis from one swine farm to another
Source: Parasit Vectors. 2021 Jul 9;14:359. doi: 10.1186/s13071-021-04861-9 (PMC8268521; doi:10.1186/s13071-021-04861-9)
Supplement: Supplementary file 5 — Additional file 5. Inferred Q percentage of membership of each individual for different values of k. [file 13071_2021_4861_MOESM5_ESM.pdf]

Additional file 5 - Inferred Q percentage of membership of each individual for different values of k. A, graphical representation of the percentages of individuals assigned to a specific cluster with a Q % of membership ≥ 0.7. The colors represent the different clusters hypothesized for that simulation; grey sector represent the percentage of admixed individuals. B, raw Q values computed by STRUCTURE and used to draw panel A, colored background highlights values ≥ 0.7.

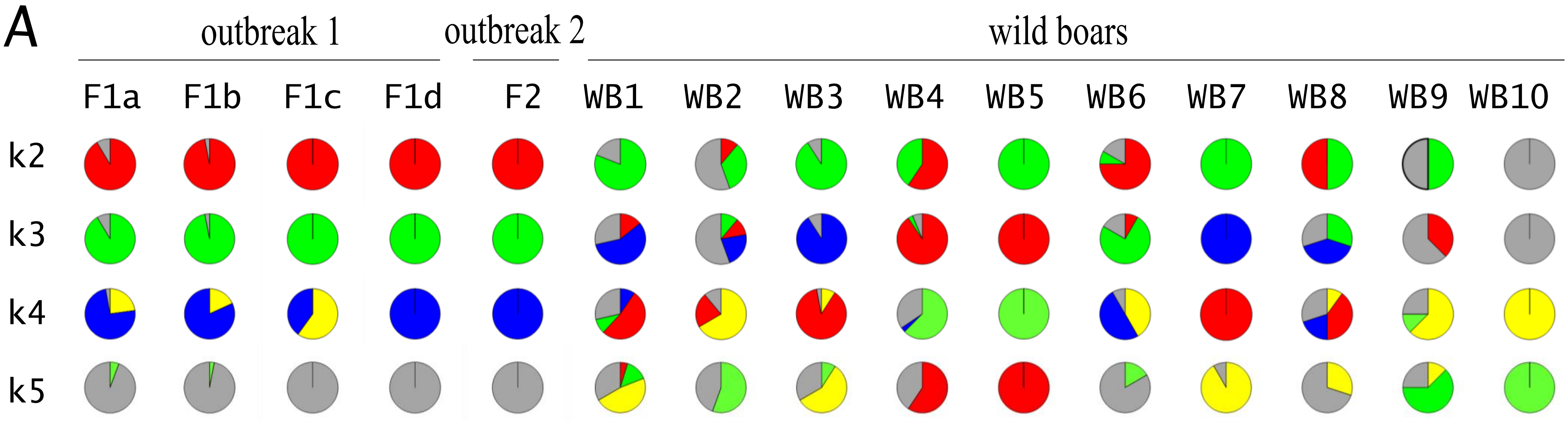

B

	k1	k2	k1	k2	k3	k1	k2	k3	k4	k1	k2	k3	k4	k5
F1a-01	0.99	0.02	0.01	0.97	0.02	0.12	0.01	0.01	0.85	0.01	0.02	0.48	0.01	0.48
F1a-02	0.98	0.02	0.01	0.97	0.02	0.13	0.01	0.01	0.85	0.01	0.02	0.49	0.01	0.47
F1a-03	0.99	0.02	0.01	0.97	0.02	0.12	0.01	0.01	0.86	0.01	0.02	0.48	0.01	0.47
F1a-04	0.99	0.02	0.01	0.97	0.02	0.13	0.01	0.01	0.85	0.01	0.02	0.48	0.01	0.47
F1a-05	0.98	0.02	0.01	0.97	0.02	0.12	0.01	0.01	0.86	0.01	0.02	0.48	0.01	0.48
F1a-06	0.99	0.02	0.01	0.97	0.02	0.13	0.01	0.01	0.85	0.01	0.02	0.48	0.01	0.48
F1a-07	0.99	0.02	0.01	0.97	0.02	0.12	0.01	0.01	0.86	0.01	0.02	0.48	0.01	0.48
F1a-08	0.98	0.02	0.01	0.97	0.02	0.12	0.01	0.01	0.85	0.01	0.03	0.48	0.01	0.48
F1a-09	0.99	0.02	0.01	0.97	0.02	0.12	0.01	0.01	0.86	0.01	0.02	0.48	0.01	0.48
F1a-10	0.99	0.02	0.01	0.97	0.02	0.13	0.01	0.01	0.85	0.01	0.03	0.48	0.01	0.47
F1a-11	0.98	0.02	0.02	0.96	0.02	0.13	0.01	0.01	0.85	0.01	0.02	0.48	0.01	0.47
F1a-12	0.99	0.02	0.01	0.97	0.02	0.13	0.01	0.01	0.85	0.01	0.02	0.47	0.01	0.48
F1a-13	0.92	0.08	0.09	0.81	0.10	0.74	0.04	0.03	0.20	0.02	0.22	0.36	0.03	0.37
F1a-14	0.98	0.02	0.01	0.97	0.02	0.12	0.01	0.01	0.85	0.01	0.03	0.48	0.01	0.47
F1a-15	0.99	0.02	0.01	0.97	0.02	0.12	0.01	0.01	0.85	0.01	0.02	0.48	0.01	0.48
F1a-16	0.99	0.02	0.01	0.97	0.02	0.12	0.01	0.01	0.86	0.01	0.03	0.48	0.01	0.47
F1a-17	0.99	0.02	0.01	0.97	0.02	0.12	0.01	0.01	0.85	0.01	0.02	0.47	0.01	0.48
F1a-18	0.93	0.07	0.08	0.82	0.10	0.73	0.04	0.03	0.20	0.02	0.22	0.36	0.03	0.37
F1a-19	0.93	0.07	0.08	0.82	0.10	0.74	0.04	0.03	0.20	0.03	0.22	0.36	0.03	0.37
F1a-20	0.93	0.07	0.08	0.81	0.11	0.74	0.04	0.03	0.19	0.02	0.22	0.36	0.03	0.37
F1a-21	0.41	0.59	0.49	0.24	0.27	0.85	0.08	0.03	0.04	0.02	0.81	0.07	0.04	0.06
F1a-22	0.42	0.58	0.63	0.31	0.06	0.44	0.35	0.03	0.18	0.02	0.19	0.26	0.27	0.26
F1a-23	0.92	0.08	0.10	0.78	0.12	0.73	0.04	0.04	0.20	0.03	0.21	0.37	0.03	0.37
F1a-24	0.98	0.02	0.01	0.97	0.02	0.12	0.01	0.01	0.86	0.01	0.02	0.48	0.01	0.47
F1a-25	0.41	0.59	0.49	0.24	0.27	0.86	0.08	0.03	0.03	0.02	0.81	0.06	0.04	0.06
F1a-26	0.98	0.02	0.01	0.97	0.02	0.12	0.01	0.01	0.86	0.01	0.03	0.48	0.01	0.48
F1a-27	0.93	0.07	0.08	0.82	0.10	0.73	0.04	0.03	0.20	0.02	0.22	0.36	0.03	0.37
F1a-28	0.99	0.02	0.01	0.97	0.02	0.13	0.01	0.01	0.85	0.01	0.02	0.47	0.01	0.48
F1a-29	0.99	0.02	0.01	0.97	0.02	0.12	0.01	0.01	0.85	0.01	0.03	0.48	0.01	0.48
F1a-30	0.98	0.02	0.01	0.97	0.02	0.13	0.01	0.01	0.85	0.01	0.03	0.48	0.01	0.47
F1a-31	0.98	0.02	0.01	0.97	0.02	0.12	0.01	0.01	0.86	0.01	0.02	0.47	0.01	0.48
F1a-32	0.98	0.02	0.01	0.97	0.02	0.13	0.01	0.01	0.85	0.01	0.02	0.48	0.01	0.48
F1a-33	0.99	0.02	0.01	0.97	0.02	0.13	0.01	0.01	0.85	0.01	0.02	0.49	0.01	0.47
F1a-34	0.98	0.02	0.01	0.97	0.02	0.13	0.01	0.01	0.85	0.01	0.03	0.48	0.01	0.48
F1a-35	0.99	0.02	0.01	0.97	0.02	0.13	0.01	0.01	0.85	0.01	0.03	0.48	0.01	0.48
F1b-01	0.98	0.02	0.02	0.96	0.02	0.13	0.01	0.01	0.85	0.01	0.02	0.49	0.01	0.47
F1b-02	0.99	0.02	0.02	0.97	0.02	0.12	0.01	0.01	0.86	0.01	0.03	0.48	0.01	0.48
F1b-03	0.98	0.02	0.01	0.97	0.02	0.13	0.01	0.01	0.85	0.01	0.03	0.48	0.01	0.48
F1b-04	0.93	0.07	0.09	0.81	0.10	0.74	0.04	0.03	0.20	0.02	0.22	0.36	0.03	0.36
F1b-05	0.99	0.02	0.01	0.97	0.02	0.12	0.01	0.01	0.86	0.01	0.03	0.48	0.01	0.48
F1b-06	0.98	0.02	0.02	0.96	0.03	0.22	0.01	0.02	0.75	0.01	0.03	0.48	0.01	0.47
F1b-07	0.98	0.02	0.01	0.97	0.02	0.12	0.01	0.01	0.86	0.01	0.03	0.48	0.01	0.48
F1b-08	0.99	0.02	0.01	0.97	0.02	0.13	0.01	0.01	0.85	0.01	0.02	0.48	0.01	0.48
F1b-09	0.99	0.02	0.02	0.97	0.02	0.12	0.01	0.01	0.85	0.01	0.02	0.48	0.01	0.47
F1b-10	0.98	0.02	0.01	0.97	0.02	0.12	0.01	0.01	0.86	0.01	0.03	0.48	0.01	0.48
F1b-11	0.98	0.02	0.01	0.97	0.02	0.12	0.01	0.01	0.86	0.01	0.02	0.48	0.01	0.48
F1b-12	0.98	0.02	0.01	0.97	0.02	0.13	0.01	0.01	0.85	0.01	0.02	0.49	0.01	0.47
F1b-13	0.98	0.02	0.01	0.97	0.02	0.12	0.01	0.01	0.86	0.01	0.03	0.48	0.01	0.48
F1b-14	0.99	0.02	0.01	0.97	0.02	0.13	0.01	0.01	0.85	0.01	0.02	0.47	0.01	0.48

F1b-15	0.99	0.02	0.01	0.97	0.02	0.12	0.01	0.01	0.86	0.01	0.03	0.48	0.01	0.47
F1b-16	0.41	0.59	0.49	0.24	0.27	0.85	0.08	0.03	0.04	0.02	0.81	0.07	0.04	0.07
F1b-17	0.99	0.02	0.01	0.97	0.02	0.13	0.01	0.01	0.85	0.01	0.02	0.48	0.01	0.47
F1b-18	0.92	0.08	0.08	0.82	0.10	0.73	0.04	0.03	0.20	0.02	0.22	0.36	0.03	0.36
F1b-19	0.93	0.08	0.08	0.82	0.10	0.74	0.04	0.03	0.20	0.02	0.22	0.35	0.03	0.37
F1b-20	0.99	0.02	0.01	0.97	0.02	0.12	0.01	0.01	0.86	0.01	0.03	0.48	0.01	0.48
F1b-21	0.93	0.08	0.09	0.81	0.10	0.74	0.04	0.03	0.20	0.02	0.23	0.36	0.03	0.36
F1b-22	0.98	0.02	0.01	0.97	0.02	0.12	0.01	0.01	0.85	0.01	0.03	0.48	0.01	0.47
F1b-23	0.99	0.02	0.01	0.97	0.02	0.13	0.01	0.01	0.85	0.01	0.03	0.48	0.01	0.47
F1b-24	0.98	0.02	0.01	0.97	0.02	0.13	0.01	0.01	0.85	0.01	0.02	0.48	0.01	0.48
F1b-25	0.99	0.02	0.01	0.97	0.02	0.12	0.01	0.01	0.86	0.01	0.03	0.48	0.01	0.48
F1b-26	0.98	0.02	0.01	0.97	0.02	0.13	0.01	0.01	0.85	0.01	0.02	0.47	0.01	0.48
F1b-27	0.99	0.02	0.01	0.97	0.02	0.13	0.01	0.01	0.85	0.01	0.03	0.48	0.01	0.48
F1b-28	0.98	0.02	0.01	0.97	0.02	0.13	0.01	0.01	0.85	0.01	0.02	0.49	0.01	0.47
F1b-29	0.98	0.02	0.01	0.97	0.02	0.13	0.01	0.01	0.85	0.01	0.02	0.48	0.01	0.48
F1b-30	0.93	0.08	0.08	0.82	0.10	0.73	0.04	0.03	0.20	0.02	0.22	0.36	0.03	0.36
F1b-31	0.98	0.02	0.01	0.97	0.02	0.13	0.01	0.01	0.85	0.01	0.03	0.48	0.01	0.47
F1b-32	0.99	0.02	0.01	0.97	0.02	0.13	0.01	0.01	0.85	0.01	0.02	0.48	0.01	0.48
F1b-33	0.98	0.02	0.01	0.97	0.02	0.13	0.01	0.01	0.85	0.01	0.03	0.48	0.01	0.47
F1c-01	0.93	0.08	0.09	0.81	0.10	0.74	0.04	0.03	0.19	0.02	0.22	0.36	0.03	0.36
F1c-02	0.92	0.08	0.08	0.82	0.10	0.74	0.04	0.03	0.20	0.03	0.23	0.36	0.03	0.36
F1c-03	0.93	0.08	0.08	0.81	0.10	0.73	0.04	0.03	0.20	0.02	0.23	0.36	0.03	0.36
F1c-04	0.99	0.02	0.01	0.97	0.02	0.12	0.01	0.01	0.86	0.01	0.03	0.48	0.01	0.47
F1c-05	0.92	0.08	0.08	0.82	0.10	0.74	0.04	0.03	0.19	0.02	0.22	0.36	0.03	0.36
F1c-06	0.92	0.08	0.08	0.81	0.10	0.74	0.04	0.03	0.20	0.02	0.23	0.36	0.03	0.36
F1c-07	0.93	0.07	0.08	0.82	0.10	0.74	0.04	0.03	0.20	0.02	0.23	0.36	0.03	0.36
F1c-08	0.99	0.02	0.01	0.97	0.02	0.12	0.01	0.01	0.85	0.01	0.03	0.48	0.01	0.48
F1c-09	0.93	0.07	0.08	0.81	0.10	0.74	0.04	0.03	0.20	0.02	0.22	0.36	0.03	0.36
F1c-10	0.93	0.08	0.09	0.81	0.10	0.74	0.04	0.03	0.20	0.02	0.22	0.36	0.03	0.36
F1c-11	0.98	0.02	0.01	0.97	0.02	0.13	0.01	0.01	0.85	0.01	0.02	0.48	0.01	0.48
F1c-12	0.99	0.02	0.01	0.97	0.02	0.13	0.01	0.01	0.85	0.01	0.03	0.48	0.01	0.48
F1c-13	0.90	0.10	0.13	0.78	0.10	0.72	0.06	0.03	0.19	0.02	0.22	0.36	0.05	0.36
F1c-14	0.93	0.08	0.08	0.82	0.10	0.74	0.04	0.03	0.20	0.02	0.22	0.36	0.03	0.37
F1c-15	0.99	0.02	0.01	0.97	0.02	0.13	0.01	0.01	0.85	0.01	0.02	0.48	0.01	0.48
F1c-16	0.99	0.02	0.01	0.97	0.02	0.13	0.01	0.01	0.85	0.01	0.02	0.48	0.01	0.48
F1c-17	0.93	0.07	0.08	0.81	0.10	0.74	0.04	0.03	0.20	0.02	0.22	0.36	0.03	0.37
F1c-18	0.93	0.07	0.08	0.82	0.10	0.74	0.04	0.03	0.20	0.02	0.22	0.36	0.03	0.37
F1c-19	0.93	0.07	0.08	0.82	0.10	0.74	0.04	0.03	0.19	0.02	0.22	0.36	0.03	0.37
F1c-20	0.99	0.02	0.01	0.97	0.02	0.12	0.01	0.01	0.86	0.01	0.02	0.48	0.01	0.47
F1c-21	0.98	0.02	0.01	0.97	0.02	0.12	0.01	0.01	0.86	0.01	0.03	0.48	0.01	0.47
F1c-22	0.98	0.02	0.01	0.97	0.02	0.13	0.01	0.01	0.85	0.01	0.03	0.48	0.01	0.48
F1c-23	0.98	0.02	0.01	0.97	0.02	0.13	0.01	0.01	0.85	0.01	0.02	0.48	0.01	0.47
F1c-24	0.99	0.02	0.01	0.97	0.02	0.13	0.01	0.01	0.85	0.01	0.02	0.48	0.01	0.48
F1c-25	0.93	0.07	0.08	0.81	0.10	0.75	0.04	0.03	0.19	0.02	0.22	0.36	0.03	0.36
F1c-26	0.93	0.08	0.08	0.81	0.10	0.74	0.04	0.03	0.19	0.02	0.22	0.36	0.03	0.37
F1c-27	0.93	0.07	0.08	0.81	0.10	0.74	0.04	0.03	0.20	0.02	0.22	0.35	0.03	0.37
F1c-28	0.93	0.08	0.09	0.81	0.10	0.74	0.04	0.03	0.20	0.02	0.22	0.36	0.03	0.37
F1c-29	0.93	0.07	0.08	0.82	0.10	0.74	0.04	0.03	0.20	0.02	0.23	0.35	0.03	0.37
F1c-30	0.98	0.02	0.01	0.97	0.02	0.13	0.01	0.01	0.85	0.01	0.02	0.48	0.01	0.48
F1c-31	0.93	0.08	0.09	0.81	0.10	0.74	0.04	0.03	0.20	0.02	0.22	0.36	0.03	0.37
F1c-32	0.93	0.07	0.10	0.82	0.08	0.74	0.04	0.03	0.20	0.02	0.22	0.36	0.03	0.37
F1c-33	0.98	0.02	0.02	0.97	0.01	0.13	0.01	0.01	0.85	0.01	0.03	0.48	0.01	0.47
F1c-34	0.93	0.07	0.11	0.81	0.09	0.74	0.04	0.03	0.20	0.03	0.22	0.36	0.03	0.37
F1c-35	0.98	0.02	0.02	0.96	0.02	0.13	0.02	0.01	0.84	0.01	0.03	0.48	0.02	0.47
F1d-01	0.98	0.02	0.02	0.97	0.01	0.12	0.01	0.01	0.86	0.01	0.02	0.48	0.01	0.48
F1d-02	0.98	0.02	0.02	0.97	0.01	0.13	0.01	0.01	0.85	0.01	0.02	0.48	0.01	0.48
F1d-03	0.98	0.02	0.02	0.97	0.01	0.13	0.01	0.01	0.85	0.01	0.02	0.48	0.01	0.48
F1d-04	0.98	0.02	0.02	0.97	0.01	0.12	0.01	0.01	0.86	0.01	0.03	0.48	0.01	0.47
F1d-05	0.99	0.02	0.02	0.97	0.01	0.13	0.01	0.01	0.85	0.01	0.02	0.48	0.01	0.47
F1d-06	0.98	0.02	0.02	0.97	0.01	0.12	0.01	0.01	0.86	0.01	0.03	0.48	0.01	0.47
F1d-07	0.99	0.02	0.02	0.97	0.01	0.12	0.01	0.01	0.86	0.01	0.02	0.48	0.01	0.47
F1d-08	0.98	0.02	0.02	0.97	0.01	0.13	0.01	0.01	0.85	0.01	0.02	0.48	0.01	0.48
F1d-09	0.99	0.02	0.02	0.97	0.01	0.12	0.01	0.01	0.86	0.01	0.02	0.48	0.01	0.48
F2-01	0.99	0.02	0.02	0.97	0.01	0.13	0.01	0.01	0.85	0.01	0.03	0.48	0.01	0.47
F2-02	0.98	0.02	0.02	0.97	0.01	0.13	0.01	0.01	0.85	0.01	0.02	0.48	0.01	0.48
F2-03	0.99	0.02	0.02	0.97	0.01	0.12	0.01	0.01	0.86	0.01	0.03	0.48	0.01	0.47
F2-04	0.99	0.02	0.02	0.97	0.01	0.13	0.01	0.01	0.85	0.01	0.03	0.48	0.01	0.47
F2-05	0.98	0.02	0.02	0.97	0.01	0.12	0.01	0.01	0.86	0.01	0.02	0.48	0.01	0.47
F2-06	0.99	0.02	0.02	0.97	0.01	0.12	0.01	0.01	0.86	0.01	0.02	0.47	0.01	0.48

F2-07	0.99	0.02	0.02	0.97	0.01	0.12	0.01	0.01	0.85	0.01	0.02	0.48	0.01	0.47
F2-08	0.99	0.02	0.02	0.97	0.02	0.13	0.01	0.01	0.85	0.01	0.02	0.48	0.01	0.48
F2-09	0.99	0.02	0.02	0.97	0.01	0.12	0.01	0.01	0.86	0.01	0.03	0.48	0.01	0.47
F2-10	0.99	0.02	0.02	0.97	0.01	0.12	0.01	0.01	0.86	0.01	0.02	0.48	0.01	0.48
F2-11	0.98	0.02	0.02	0.97	0.01	0.12	0.01	0.01	0.86	0.01	0.02	0.48	0.01	0.47
WB1-01	0.04	0.96	0.12	0.03	0.85	0.19	0.72	0.06	0.03	0.03	0.46	0.04	0.44	0.04
WB1-02	0.10	0.90	0.25	0.07	0.68	0.66	0.25	0.06	0.03	0.02	0.82	0.04	0.08	0.04
WB1-03	0.03	0.97	0.02	0.02	0.96	0.03	0.93	0.02	0.03	0.02	0.03	0.03	0.89	0.03
WB1-04	0.01	0.99	0.02	0.01	0.97	0.02	0.96	0.01	0.01	0.01	0.03	0.01	0.93	0.01
WB1-05	0.41	0.59	0.28	0.24	0.48	0.85	0.08	0.03	0.04	0.02	0.81	0.07	0.04	0.07
WB1-06	0.56	0.44	0.12	0.41	0.46	0.58	0.22	0.05	0.16	0.04	0.26	0.28	0.15	0.28
WB1-07	0.32	0.68	0.91	0.07	0.02	0.04	0.03	0.80	0.13	0.68	0.03	0.13	0.03	0.13
WB1-08	0.08	0.92	0.94	0.03	0.03	0.04	0.05	0.87	0.05	0.77	0.05	0.06	0.06	0.06
WB1-09	0.02	0.98	0.02	0.02	0.96	0.04	0.92	0.02	0.02	0.02	0.10	0.03	0.84	0.02
WB1-10	0.29	0.72	0.19	0.19	0.62	0.31	0.45	0.08	0.16	0.06	0.22	0.19	0.34	0.19
WB1-11	0.02	0.98	0.03	0.02	0.95	0.06	0.90	0.02	0.02	0.02	0.19	0.03	0.73	0.03
WB1-12	0.41	0.60	0.28	0.24	0.48	0.86	0.08	0.03	0.03	0.02	0.80	0.07	0.04	0.07
WB1-13	0.01	0.99	0.01	0.01	0.98	0.02	0.96	0.01	0.01	0.01	0.04	0.01	0.93	0.01
WB1-14	0.01	0.99	0.13	0.01	0.86	0.02	0.84	0.14	0.01	0.14	0.05	0.01	0.79	0.01
WB1-15	0.02	0.98	0.64	0.03	0.33	0.14	0.43	0.40	0.03	0.26	0.36	0.04	0.31	0.04
WB1-16	0.01	0.99	0.01	0.01	0.98	0.02	0.97	0.01	0.01	0.01	0.02	0.01	0.95	0.01
WB1-17	0.03	0.97	0.02	0.02	0.96	0.08	0.88	0.02	0.03	0.02	0.17	0.04	0.75	0.04
WB1-18	0.03	0.97	0.83	0.02	0.15	0.10	0.19	0.69	0.03	0.42	0.36	0.03	0.16	0.03
WB1-19	0.02	0.98	0.04	0.02	0.95	0.03	0.94	0.02	0.01	0.02	0.07	0.02	0.88	0.02
WB1-20	0.02	0.98	0.02	0.02	0.96	0.04	0.92	0.02	0.02	0.02	0.10	0.03	0.84	0.02
WB1-21	0.02	0.98	0.11	0.02	0.87	0.29	0.62	0.07	0.02	0.03	0.69	0.02	0.23	0.03
WB2-01	0.41	0.59	0.27	0.24	0.49	0.86	0.08	0.03	0.03	0.02	0.81	0.06	0.04	0.07
WB2-02	0.12	0.89	0.06	0.09	0.86	0.11	0.75	0.04	0.09	0.03	0.11	0.11	0.64	0.11
WB2-03	0.93	0.08	0.10	0.82	0.08	0.74	0.04	0.03	0.19	0.02	0.22	0.36	0.03	0.37
WB2-04	0.41	0.59	0.28	0.24	0.47	0.86	0.08	0.03	0.03	0.02	0.81	0.06	0.04	0.07
WB2-05	0.41	0.59	0.29	0.24	0.48	0.86	0.08	0.03	0.03	0.02	0.81	0.06	0.04	0.07
WB2-06	0.04	0.96	0.12	0.03	0.85	0.19	0.72	0.06	0.03	0.03	0.46	0.04	0.44	0.04
WB2-07	0.07	0.93	0.80	0.07	0.13	0.53	0.09	0.34	0.05	0.17	0.66	0.06	0.05	0.06
WB2-08	0.41	0.59	0.28	0.25	0.48	0.86	0.07	0.03	0.03	0.02	0.81	0.07	0.04	0.07
WB2-09	0.41	0.59	0.28	0.24	0.48	0.86	0.08	0.03	0.04	0.02	0.81	0.07	0.04	0.07
WB3-01	0.05	0.95	0.13	0.03	0.84	0.19	0.72	0.06	0.03	0.04	0.45	0.04	0.44	0.04
WB3-02	0.41	0.59	0.28	0.25	0.47	0.86	0.08	0.04	0.04	0.02	0.80	0.07	0.04	0.07
WB3-03	0.02	0.98	0.02	0.02	0.96	0.04	0.92	0.02	0.02	0.02	0.10	0.03	0.84	0.03
WB3-04	0.04	0.96	0.12	0.03	0.85	0.19	0.72	0.05	0.03	0.04	0.45	0.04	0.44	0.04
WB3-05	0.41	0.59	0.28	0.24	0.48	0.86	0.07	0.03	0.04	0.02	0.81	0.06	0.04	0.07
WB3-06	0.04	0.96	0.12	0.03	0.85	0.19	0.72	0.06	0.03	0.04	0.46	0.04	0.44	0.04
WB3-07	0.02	0.98	0.03	0.02	0.95	0.06	0.90	0.02	0.02	0.02	0.19	0.03	0.73	0.03
WB3-08	0.02	0.98	0.03	0.02	0.95	0.06	0.90	0.03	0.02	0.02	0.19	0.03	0.74	0.03
WB3-09	0.01	0.99	0.02	0.01	0.97	0.02	0.96	0.01	0.01	0.01	0.03	0.01	0.93	0.01
WB3-10	0.04	0.96	0.12	0.03	0.85	0.19	0.72	0.06	0.03	0.03	0.45	0.04	0.44	0.04
WB3-11	0.02	0.98	0.02	0.02	0.96	0.05	0.92	0.02	0.02	0.02	0.10	0.03	0.83	0.03
WB3-12	0.02	0.98	0.04	0.02	0.94	0.03	0.94	0.02	0.01	0.02	0.08	0.02	0.87	0.02
WB3-13	0.02	0.98	0.02	0.02	0.96	0.04	0.92	0.02	0.02	0.02	0.10	0.02	0.83	0.03
WB3-14	0.01	0.99	0.01	0.01	0.98	0.02	0.97	0.01	0.01	0.01	0.02	0.01	0.95	0.01
WB3-15	0.04	0.96	0.12	0.04	0.85	0.20	0.72	0.06	0.03	0.04	0.46	0.04	0.43	0.04
WB3-16	0.02	0.98	0.03	0.02	0.95	0.06	0.90	0.02	0.02	0.02	0.20	0.03	0.73	0.03
WB3-17	0.03	0.97	0.02	0.02	0.96	0.08	0.89	0.02	0.02	0.01	0.14	0.02	0.80	0.02
WB3-18	0.41	0.59	0.28	0.24	0.48	0.85	0.08	0.04	0.04	0.02	0.81	0.06	0.04	0.07
WB3-19	0.01	0.99	0.02	0.01	0.97	0.02	0.96	0.01	0.01	0.01	0.03	0.01	0.93	0.01
WB3-20	0.02	0.98	0.04	0.02	0.95	0.03	0.94	0.02	0.01	0.02	0.07	0.02	0.88	0.02
WB3-21	0.04	0.96	0.08	0.03	0.90	0.12	0.82	0.04	0.03	0.03	0.30	0.03	0.61	0.03
WB3-22	0.01	0.99	0.01	0.01	0.98	0.02	0.97	0.01	0.01	0.01	0.02	0.01	0.95	0.01
WB3-23	0.01	0.99	0.02	0.01	0.97	0.02	0.96	0.01	0.01	0.01	0.03	0.01	0.93	0.01
WB3-24	0.02	0.98	0.04	0.02	0.94	0.03	0.94	0.02	0.02	0.02	0.07	0.02	0.88	0.02
WB3-25	0.02	0.98	0.02	0.02	0.96	0.04	0.92	0.02	0.02	0.02	0.10	0.02	0.84	0.03
WB3-26	0.01	0.99	0.01	0.01	0.98	0.02	0.97	0.01	0.01	0.01	0.02	0.01	0.95	0.01
WB3-27	0.04	0.96	0.11	0.03	0.85	0.19	0.72	0.06	0.03	0.04	0.46	0.04	0.43	0.04
WB3-28	0.04	0.96	0.11	0.03	0.85	0.20	0.72	0.06	0.03	0.03	0.45	0.04	0.44	0.04
WB3-29	0.06	0.94	0.05	0.05	0.91	0.33	0.61	0.03	0.04	0.02	0.49	0.06	0.38	0.06
WB3-30	0.01	0.99	0.01	0.01	0.98	0.02	0.97	0.01	0.01	0.01	0.02	0.01	0.95	0.01
WB3-31	0.01	0.99	0.01	0.01	0.99	0.01	0.98	0.01	0.01	0.01	0.01	0.01	0.98	0.01
WB3-32	0.05	0.96	0.11	0.03	0.86	0.19	0.72	0.06	0.03	0.04	0.45	0.04	0.44	0.04
WB3-33	0.04	0.96	0.04	0.02	0.94	0.14	0.82	0.02	0.02	0.02	0.30	0.03	0.63	0.03
WB4-01	0.96	0.04	0.95	0.03	0.01	0.04	0.01	0.91	0.04	0.86	0.04	0.04	0.01	0.04

WB4-02	0.71	0.29	0.89	0.10	0.01	0.04	0.01	0.82	0.13	0.73	0.02	0.12	0.01	0.12
WB4-03	0.96	0.04	0.89	0.10	0.01	0.04	0.01	0.82	0.13	0.73	0.02	0.12	0.01	0.12
WB4-04	0.96	0.04	0.49	0.49	0.02	0.16	0.01	0.26	0.57	0.16	0.05	0.39	0.01	0.39
WB4-05	0.98	0.02	0.95	0.03	0.01	0.04	0.01	0.92	0.04	0.87	0.04	0.04	0.01	0.04
WB4-06	0.98	0.02	0.89	0.10	0.01	0.04	0.01	0.82	0.13	0.74	0.02	0.12	0.01	0.12
WB4-07	0.96	0.04	0.89	0.10	0.01	0.04	0.01	0.82	0.13	0.74	0.02	0.12	0.01	0.11
WB4-08	0.71	0.29	0.89	0.10	0.01	0.04	0.01	0.82	0.13	0.73	0.02	0.12	0.01	0.12
WB4-09	0.98	0.02	0.97	0.01	0.02	0.04	0.01	0.94	0.01	0.86	0.09	0.02	0.01	0.02
WB4-10	0.96	0.04	0.97	0.02	0.02	0.04	0.01	0.94	0.01	0.86	0.09	0.02	0.01	0.02
WB4-11	0.96	0.04	0.90	0.04	0.06	0.36	0.05	0.56	0.04	0.24	0.66	0.04	0.03	0.04
WB4-12	0.96	0.04	0.91	0.04	0.05	0.36	0.05	0.56	0.04	0.24	0.64	0.04	0.03	0.04
WB4-13	0.13	0.87	0.87	0.03	0.10	0.26	0.13	0.58	0.03	0.29	0.55	0.04	0.09	0.04
WB4-14	0.14	0.86	0.91	0.04	0.05	0.37	0.04	0.56	0.04	0.24	0.66	0.04	0.03	0.04
WB4-15	0.13	0.87	0.96	0.01	0.03	0.02	0.03	0.95	0.01	0.91	0.04	0.01	0.03	0.01
WB4-16	0.24	0.77	0.82	0.15	0.03	0.34	0.03	0.50	0.13	0.33	0.25	0.21	0.02	0.20
WB4-17	0.24	0.76	0.89	0.10	0.01	0.04	0.01	0.82	0.13	0.74	0.02	0.12	0.01	0.12
WB4-18	0.10	0.90	0.96	0.03	0.01	0.04	0.01	0.92	0.04	0.87	0.04	0.04	0.01	0.04
WB4-19	0.24	0.76	0.91	0.04	0.05	0.37	0.05	0.55	0.04	0.24	0.65	0.04	0.03	0.04
WB4-20	0.02	0.98	0.97	0.02	0.02	0.04	0.01	0.94	0.01	0.86	0.09	0.02	0.01	0.02
WB4-21	0.85	0.15	0.89	0.10	0.01	0.04	0.01	0.82	0.13	0.73	0.02	0.12	0.01	0.12
WB4-22	0.71	0.29	0.91	0.04	0.06	0.36	0.05	0.56	0.04	0.24	0.66	0.04	0.03	0.04
WB4-23	0.24	0.76	0.91	0.04	0.05	0.36	0.05	0.56	0.04	0.24	0.65	0.04	0.03	0.04
WB4-24	0.13	0.87	0.02	0.97	0.01	0.13	0.01	0.01	0.85	0.01	0.03	0.48	0.01	0.48
WB4-25	0.24	0.76	0.96	0.03	0.01	0.04	0.01	0.92	0.04	0.87	0.04	0.04	0.01	0.04
WB4-26	0.23	0.77	0.82	0.15	0.03	0.35	0.03	0.49	0.13	0.32	0.25	0.20	0.02	0.20
WB4-27	0.71	0.29	0.95	0.03	0.01	0.04	0.01	0.91	0.04	0.87	0.04	0.04	0.01	0.04
WB4-28	0.85	0.15	0.49	0.49	0.02	0.16	0.01	0.25	0.57	0.16	0.05	0.39	0.01	0.39
WB4-29	0.71	0.29	0.95	0.03	0.01	0.04	0.01	0.91	0.04	0.87	0.04	0.04	0.01	0.04
WB4-30	0.71	0.29	0.87	0.05	0.08	0.11	0.10	0.71	0.08	0.55	0.15	0.10	0.11	0.10
WB4-31	0.19	0.81	0.97	0.02	0.02	0.04	0.01	0.94	0.01	0.86	0.09	0.02	0.01	0.02
WB4-32	0.71	0.29	0.96	0.03	0.01	0.04	0.01	0.91	0.03	0.86	0.04	0.04	0.01	0.04
WB5-01	0.01	0.99	0.98	0.01	0.01	0.01	0.01	0.97	0.01	0.96	0.01	0.01	0.01	0.01
WB5-02	0.02	0.98	0.98	0.01	0.01	0.01	0.01	0.97	0.01	0.96	0.01	0.01	0.01	0.01
WB5-03	0.01	0.99	0.97	0.01	0.02	0.01	0.02	0.97	0.01	0.96	0.01	0.01	0.02	0.01
WB5-04	0.01	0.99	0.98	0.01	0.01	0.01	0.01	0.97	0.01	0.96	0.01	0.01	0.01	0.01
WB5-05	0.01	0.99	0.98	0.01	0.01	0.01	0.01	0.97	0.01	0.96	0.01	0.01	0.01	0.01
WB5-06	0.01	0.99	0.97	0.01	0.02	0.01	0.02	0.97	0.01	0.96	0.01	0.01	0.02	0.01
WB5-07	0.01	0.99	0.97	0.01	0.02	0.01	0.02	0.97	0.01	0.96	0.01	0.01	0.02	0.01
WB5-08	0.01	0.99	0.97	0.01	0.02	0.01	0.02	0.97	0.01	0.96	0.01	0.01	0.02	0.01
WB5-09	0.01	0.99	0.97	0.01	0.02	0.01	0.02	0.97	0.01	0.96	0.01	0.01	0.02	0.01
WB5-10	0.01	0.99	0.97	0.01	0.02	0.01	0.02	0.97	0.01	0.96	0.01	0.01	0.02	0.01
WB6-01	0.98	0.02	0.02	0.97	0.01	0.13	0.01	0.01	0.85	0.01	0.03	0.49	0.01	0.47
WB6-02	0.41	0.59	0.27	0.24	0.49	0.86	0.08	0.03	0.04	0.02	0.81	0.06	0.04	0.07
WB6-03	0.93	0.08	0.11	0.81	0.08	0.74	0.04	0.03	0.20	0.02	0.23	0.35	0.03	0.37
WB6-04	0.98	0.02	0.02	0.97	0.01	0.12	0.01	0.01	0.86	0.01	0.02	0.47	0.01	0.48
WB6-05	0.93	0.07	0.11	0.81	0.08	0.74	0.04	0.03	0.20	0.02	0.22	0.36	0.03	0.37
WB6-06	0.99	0.02	0.02	0.97	0.01	0.13	0.01	0.01	0.85	0.01	0.03	0.48	0.01	0.47
WB6-07	0.41	0.59	0.28	0.24	0.48	0.85	0.08	0.04	0.04	0.02	0.81	0.07	0.04	0.07
WB6-08	0.08	0.92	0.89	0.03	0.08	0.29	0.06	0.62	0.03	0.25	0.67	0.03	0.03	0.03
WB6-09	0.98	0.02	0.02	0.97	0.01	0.12	0.01	0.01	0.86	0.01	0.03	0.48	0.01	0.48
WB6-10	0.99	0.02	0.02	0.97	0.01	0.13	0.01	0.01	0.85	0.01	0.03	0.48	0.01	0.48
WB6-11	0.93	0.07	0.10	0.82	0.08	0.74	0.04	0.03	0.20	0.02	0.22	0.36	0.03	0.36
WB6-12	0.98	0.02	0.02	0.97	0.01	0.13	0.01	0.01	0.85	0.01	0.02	0.48	0.01	0.48
WB7-01	0.06	0.94	0.02	0.04	0.94	0.03	0.92	0.02	0.04	0.02	0.02	0.04	0.89	0.04
WB7-02	0.12	0.88	0.02	0.08	0.91	0.02	0.88	0.01	0.09	0.01	0.01	0.07	0.85	0.07
WB7-03	0.02	0.98	0.02	0.01	0.97	0.02	0.96	0.01	0.01	0.01	0.02	0.01	0.94	0.01
WB7-04	0.03	0.97	0.01	0.02	0.97	0.02	0.95	0.01	0.02	0.01	0.01	0.02	0.94	0.02
WB7-05	0.02	0.99	0.02	0.01	0.97	0.02	0.96	0.01	0.01	0.01	0.02	0.01	0.94	0.01
WB7-06	0.03	0.97	0.01	0.02	0.97	0.02	0.95	0.01	0.02	0.01	0.01	0.02	0.94	0.02
WB7-07	0.03	0.97	0.01	0.02	0.97	0.02	0.95	0.01	0.02	0.01	0.01	0.02	0.94	0.02
WB7-08	0.02	0.99	0.02	0.01	0.98	0.02	0.96	0.01	0.01	0.01	0.02	0.01	0.94	0.01
WB7-09	0.03	0.97	0.01	0.02	0.97	0.02	0.95	0.01	0.02	0.01	0.01	0.02	0.94	0.02
WB7-10	0.01	0.99	0.01	0.01	0.98	0.01	0.98	0.01	0.01	0.01	0.01	0.01	0.97	0.01
WB7-11	0.25	0.75	0.04	0.17	0.80	0.04	0.75	0.02	0.18	0.02	0.02	0.13	0.69	0.14
WB7-12	0.12	0.88	0.02	0.08	0.91	0.02	0.88	0.01	0.09	0.01	0.01	0.07	0.85	0.07
WB8-01	0.02	0.99	0.02	0.01	0.97	0.02	0.96	0.01	0.01	0.01	0.03	0.01	0.94	0.01
WB8-02	0.97	0.03	0.02	0.94	0.04	0.16	0.03	0.01	0.80	0.01	0.02	0.47	0.02	0.47
WB8-03	0.28	0.72	0.19	0.20	0.62	0.30	0.46	0.08	0.16	0.06	0.22	0.19	0.34	0.19
WB8-04	0.79	0.21	0.07	0.64	0.29	0.60	0.15	0.03	0.22	0.02	0.16	0.35	0.10	0.36

WB8-05	0.08	0.92	0.04	0.06	0.91	0.09	0.82	0.03	0.07	0.03	0.11	0.09	0.68	0.09	
WB8-06	0.93	0.07	0.10	0.82	0.08	0.74	0.04	0.03	0.19	0.02	0.22	0.36	0.03	0.36	
WB8-07	0.02	0.98	0.04	0.02	0.95	0.03	0.94	0.02	0.01	0.02	0.07	0.02	0.88	0.02	
WB8-08	0.79	0.21	0.07	0.64	0.30	0.60	0.15	0.03	0.23	0.02	0.16	0.36	0.10	0.35	
WB8-09	0.02	0.99	0.02	0.01	0.98	0.02	0.96	0.01	0.01	0.01	0.02	0.01	0.94	0.01	
WB8-10	0.97	0.03	0.02	0.94	0.04	0.16	0.03	0.01	0.80	0.01	0.02	0.47	0.02	0.47	
WB9-01	0.04	0.96	0.28	0.24	0.48	0.86	0.08	0.03	0.04	0.02	0.81	0.07	0.04	0.07	
WB9-02	0.13	0.87	0.97	0.01	0.02	0.03	0.02	0.94	0.01	0.82	0.13	0.01	0.01	0.02	
WB9-03	0.07	0.93	0.28	0.24	0.48	0.86	0.08	0.03	0.03	0.02	0.81	0.06	0.04	0.07	
WB9-04	0.24	0.76	0.28	0.24	0.48	0.86	0.08	0.03	0.04	0.02	0.81	0.07	0.04	0.06	
WB9-05	0.41	0.59	0.91	0.03	0.07	0.28	0.06	0.64	0.03	0.25	0.66	0.03	0.03	0.03	
WB9-06	0.41	0.59	0.91	0.04	0.05	0.36	0.05	0.56	0.04	0.24	0.65	0.04	0.03	0.04	
WB9-07	0.41	0.59	0.29	0.25	0.47	0.86	0.08	0.03	0.03	0.02	0.81	0.07	0.04	0.07	
WB9-08	0.41	0.59	0.32	0.08	0.59	0.73	0.17	0.07	0.03	0.02	0.84	0.04	0.05	0.04	
WB10-01	0.41	0.59	0.28	0.25	0.48	0.85	0.08	0.04	0.04	0.02	0.81	0.06	0.04	0.07	
WB10-02	0.41	0.59	0.28	0.24	0.48	0.86	0.08	0.03	0.03	0.02	0.81	0.07	0.04	0.06	
WB10-03	0.41	0.59	0.28	0.24	0.48	0.86	0.08	0.03	0.04	0.02	0.81	0.06	0.04	0.07	
WB10-04	0.41	0.59	0.28	0.24	0.48	0.85	0.08	0.03	0.04	0.02	0.81	0.07	0.04	0.07	
WB10-05	0.41	0.59	0.28	0.24	0.48	0.85	0.08	0.03	0.04	0.02	0.80	0.07	0.04	0.07	
WB10-06	0.41	0.59	0.27	0.25	0.48	0.85	0.08	0.03	0.03	0.02	0.81	0.07	0.04	0.07	
WB10-07	0.41	0.59	0.28	0.24	0.48	0.85	0.08	0.03	0.03	0.02	0.81	0.07	0.04	0.07	
WB10-08	0.41	0.59	0.28	0.24	0.48	0.85	0.08	0.03	0.03	0.02	0.81	0.06	0.04	0.07	
WB10-09	0.41	0.59	0.28	0.24	0.48	0.85	0.08	0.03	0.04	0.02	0.81	0.07	0.04	0.07	
WB10-10	0.41	0.59	0.29	0.24	0.48	0.86	0.08	0.03	0.03	0.02	0.81	0.06	0.04	0.07	
WB10-11	0.41	0.59	0.28	0.24	0.48	0.86	0.08	0.03	0.03	0.02	0.81	0.06	0.04	0.07	
