# Supplementary material for: Genetic evidence substantiates transmission of Trichinella spiralis from one swine farm to another
Source: Parasit Vectors. 2021 Jul 9;14:359. doi: 10.1186/s13071-021-04861-9 (PMC8268521; doi:10.1186/s13071-021-04861-9)
Supplement: Supplementary file 6 — Additional file 6. When no a priori degree of subdivision can be assumed for a population, STRUCTURE affords the means to consider how biological samples cohere, genetically, assuming greater or lesser degrees of subdivision. [file 13071_2021_4861_MOESM6_ESM.docx]

Additional file 6 - When no *a priori* degree of subdivision can be assumed for a population, STRUCTURE affords the means to consider how biological samples cohere, genetically, assuming greater or lesser degrees of subdivision.

We simulated the structure of subdivisions for these larvae assuming as few as 2 or as many as 8 underlying subdivisions (Figure 3). The variation of mean posterior probabilities of "L (K)" and of the delta "K (ΔK)", for increasing values of k (as suggested by Evanno et al. [38]), determined that MLGs cannot be reliably grouped into separate membership groups / clusters (Figure 3). The “L(K)” function most favored k = 3 or k = 4. This result was not supported by the “K(ΔK)” function [38]. However, it should be noted that simulations assuming k = 4 shows Q probability values, equal to or greater than 0.7, assigning 91% of individuals to a single cluster based on our criteria (see Additional file 4, panel B). Simulations run with k = 2, 3 and 5 shown lower values of single cluster membership, 88 %, 85 % and 38 %, respectively (see Additional file 4, panels A and B).
